# JCAD deficiency delayed liver regenerative repair through the Hippo–YAP signalling pathway

**DOI:** 10.1002/ctm2.1630

**Published:** 2024-03-21

**Authors:** Li Zhang, Yong‐Yu Yang, Li Xie, Yuan Zhou, Zhenxing Zhong, Jia Ding, Zhong‐Hua Wang, Yu‐Li Wang, Xiu‐Ping Liu, Fa‐Xing Yu, Jian Wu

**Affiliations:** ^1^ Department of Medical Microbiology & Parasitology MOE/NHC/CAMS Key Laboratory of Medical Molecular Virology School of Basic Medical Sciences Fudan University Shanghai Medical College Shanghai China; ^2^ Institute of Pediatrics Children's Hospital of Fudan University Shanghai Key Laboratory of Medical Epigenetics International Co‐Laboratory of Medical Epigenetics and Metabolism Institutes of Biomedical Sciences Fudan University Shanghai Medical College Shanghai China; ^3^ Jing'an Central District Hospital Shanghai China; ^4^ Department of Pathology and Laboratory Medicine School of Basic Medical Sciences Fudan University Shanghai China; ^5^ Department of Gastroenterology & Hepatology Zhongshan Hospital of Fudan University Shanghai China; ^6^ Shanghai Institute of Liver Diseases Fudan University Shanghai Medical College Shanghai China

**Keywords:** cell cycle phase visualisation, Hippo–YAP signalling pathway, JCAD, liver regeneration, WWC1

## Abstract

**Background and aims:**

Liver regeneration retardation post partial hepatectomy (PH) is a common clinical problem after liver transplantation. Identification of key regulators in liver regeneration post PH may be beneficial for clinically improving the prognosis of patients after liver transplantation. This study aimed to clarify the function of junctional protein‐associated with coronary artery disease (JCAD) in liver regeneration post PH and to reveal the underlying mechanisms.

**Methods:**

JCAD knockout (JCAD‐KO), liver‐specific JCAD‐KO (*Jcad^△Hep^*) mice and their control group were subjected to 70% PH. RNA sequencing was conducted to unravel the related signalling pathways. Primary hepatocytes from KO mice were treated with epidermal growth factor (EGF) to evaluate DNA replication. Fluorescent ubiquitination‐based cell cycle indicator (FUCCI) live‐imaging system was used to visualise the phases of cell cycle.

**Results:**

Both global and liver‐specific JCAD deficiency postponed liver regeneration after PH as indicated by reduced gene expression of cell cycle transition and DNA replication. Prolonged retention in G1 phase and failure to transition over the cell cycle checkpoint in JCAD‐KO cell line was indicated by a FUCCI live‐imaging system as well as pharmacologic blockage. JCAD replenishment by adenovirus reversed the impaired DNA synthesis in JCAD‐KO primary hepatocyte in exposure to EGF, which was abrogated by a Yes‐associated protein (YAP) inhibitor, verteporfin. Mechanistically, JCAD competed with large tumour suppressor 2 (LATS2) for WWC1 interaction, leading to LATS2 inhibition and thereafter YAP activation, and enhanced expression of cell cycle‐associated genes.

**Conclusion:**

JCAD deficiency led to delayed regeneration after PH as a result of blockage in cell cycle progression through the Hippo–YAP signalling pathway. These findings uncovered novel functions of JCAD and suggested a potential strategy for improving graft growth and function post liver transplantation.

**Key Points:**

- JCAD deficiency leads to an impaired liver growth after PH due to cell division blockage.
- JCAD competes with LATS2 for WWC1 interaction, resulting in LATS2 inhibition, YAP activation and enhanced expression of cell cycle‐associated genes.
- Delineation of JCADHippoYAP signalling pathway would facilitate to improve prognosis of acute liver failure and graft growth in living‐donor liver transplantation.

## Graphical abstract

1. JCAD deficiency leads to an impaired liver growth after PH due to cell division blockage.
2. JCAD competes with LATS2 for WWC1 interaction, resulting in LATS2 inhibition, YAP activation and enhanced expression of cell cycle‐associated genes.
3. Delineation of JCAD–Hippo–YAP signalling pathway would facilitate to improve prognosis of acute liver failure and graft growth in living‐donor liver transplantation.

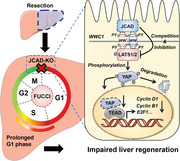

## 1 INTRODUCTION

Liver cancer, characterised by high malignancy and mortality rates, led to the second cancer‐related death in China. Surgical removal and transplant at an early stage are currently two curable options for its treatment. Liver transplantation saved thousand lives of patients with acute toxicity, genetic deficiency and end‐stage liver disease annually. The liver has a tremendous regenerative capacity following a size loss, such as partial hepatectomy (PH), through a highly orchestrated process. However, the complications of small‐for‐size syndrome following living‐donor liver transplantation (LDLT), which manifests as malfunction, loss or retarded growth of the graft, are the main causes of death in recipients.^1^ Due to extreme shortage of donor liver availability, margin grafts, such as small, split or steatotic grafts are often used in LDLT. Hundreds of LDLT has been performed successfully in the United States each year during the latest decades (www.unos.org), and globally 70% of all LDLT cases were performed in Asian countries.^2^ Unfortunately, morbidity and mortality rates are estimated to be of 23%–38% and .18%–.8%,^3^, ^4^, ^5^ partly attributed to impaired regenerative potential of the donor graft to reach the correct liver mass. Therefore, in‐depth understanding of the pathological mechanisms especially uncovering the molecular mechanisms underlying liver regeneration is crucial for improvement of graft function, quality of life and general well‐being after LDLT.

The junctional protein‐associated with coronary artery disease (JCAD), encoded by *KIAA1462*, was originally identified as a component of E‐cadherin‐based cell–cell junction. From zebrafish to humans, the sequence of JCAD is highly conserved in vertebrates indicating that the protein might have important functions in pathophysiology. The role of JCAD has been identified in pathological process of coronary artery disease^6^ as well as arterial thrombosis.^7^ Our previous work revealed that JCAD was significantly increased in steatohepatitis‐associated hepatocellular carcinoma specimens, and promoted hepatoma cells and their derived xenografts proliferation and growth.^8^ Moreover, loss of JCAD in mice resulted in impediment of tumour growth due to impaired angiogenic capacity.^9^ However, pathophysiologic role of JCAD in tissue repairing, especially in liver regeneration, has not been investigated.

Hippo–Yes‐associated protein (YAP) signalling is recognised as a crucial pathway for organ size control, tissue regeneration and stem cell self‐renewal. The canonical Hippo–YAP pathway consists of an mammalian sterile‐20‐like/large tumour suppressor (MST/LATS) kinase module and a YAP/transcriptional co‐activator with PDZ‐binding motif (TAZ)–TEA Domain Transcription Factor (TEAD) transcription module. MST1/2 and their scaffolding protein Salvador Homologue‐1 (SAV1) phosphorylate and activate LATS1/2, and this process is mediated by WWC proteins (WWC1–3).^10^ LATS1/2 together with scaffolding protein MOB1 phosphorylate YAP, which sequestrates YAP in the cytoplasm or promotes β‐Trcp‐dependent ubiquitination and degradation.^11^, ^12^ When Hippo is off, unphosphorylated YAP is translocated into the nucleus, binds to the transcription factors TEAD1–4, and transactivates target genes involved in cell proliferation and tissue growth, including connective tissue growth factor (*CTGF)*, cysteine‐rich angiogenic inducer 61 (*Cyr61*), *Ccnd1*, *E2f1* and *Birc5*.^13^, ^14^ The activity of the Hippo–YAP signalling pathway alters dynamically in accordance with liver regeneration potential. Overexpression of YAP was shown to accelerate liver regeneration after PH and toxic injury, whereas YAP inhibition yielded an opposite trend.^15^, ^16^, ^17^ Loss of mass is the original force to drive quiescent hepatocytes to enter cell cycle after PH. The Hippo signalling pathway is under exquisitely control of cell cycle and vice versa; and its signalling activity was inhibited in G1 and G1/S phase whilst activated in G2/M phase,^18^, ^19^ as validated by hyperphosphorylated YAP impeded mitosis transition. JCAD was reported to harbour two canonical ‘PPxY’ motifs, which bind strongly to WW tandems.^20^ The PPxY and WW domain‐mediated protein–protein interactions are widely present amongst Hippo pathway components, indicating that JCAD may exert its function by interacting with a protein containing WW domains in the Hippo pathway.^21^ Therefore, the present study aimed to investigate whether JCAD functions as a critical molecule in modulating liver regeneration through the Hippo–YAP signalling pathway.

In the present study, gain‐of‐function and loss‐of‐function approaches were employed to demonstrate that JCAD functions as an upstream regulator of Hippo–YAP signalling pathway and positively modulates liver regeneration. Mechanistically, JCAD competes with LATS2 for WWC1 interaction, therefore, affecting the levels of phosphorylated YAP and its kinase activity. Moreover, global and liver‐specific JCAD deficiency reduced expression of YAP‐targeted cell cycle genes and impeded liver regenerative capacity. The delineation of molecular interactions between JCAD and the Hippo–YAP signalling axis confers to develop novel therapeutic strategies in improving regenerative potential after liver failure or small‐for‐size graft transplantation.

## 2 RESULTS

### 2.1 A dynamic change of JCAD expression during liver regeneration

**FIGURE 1:**
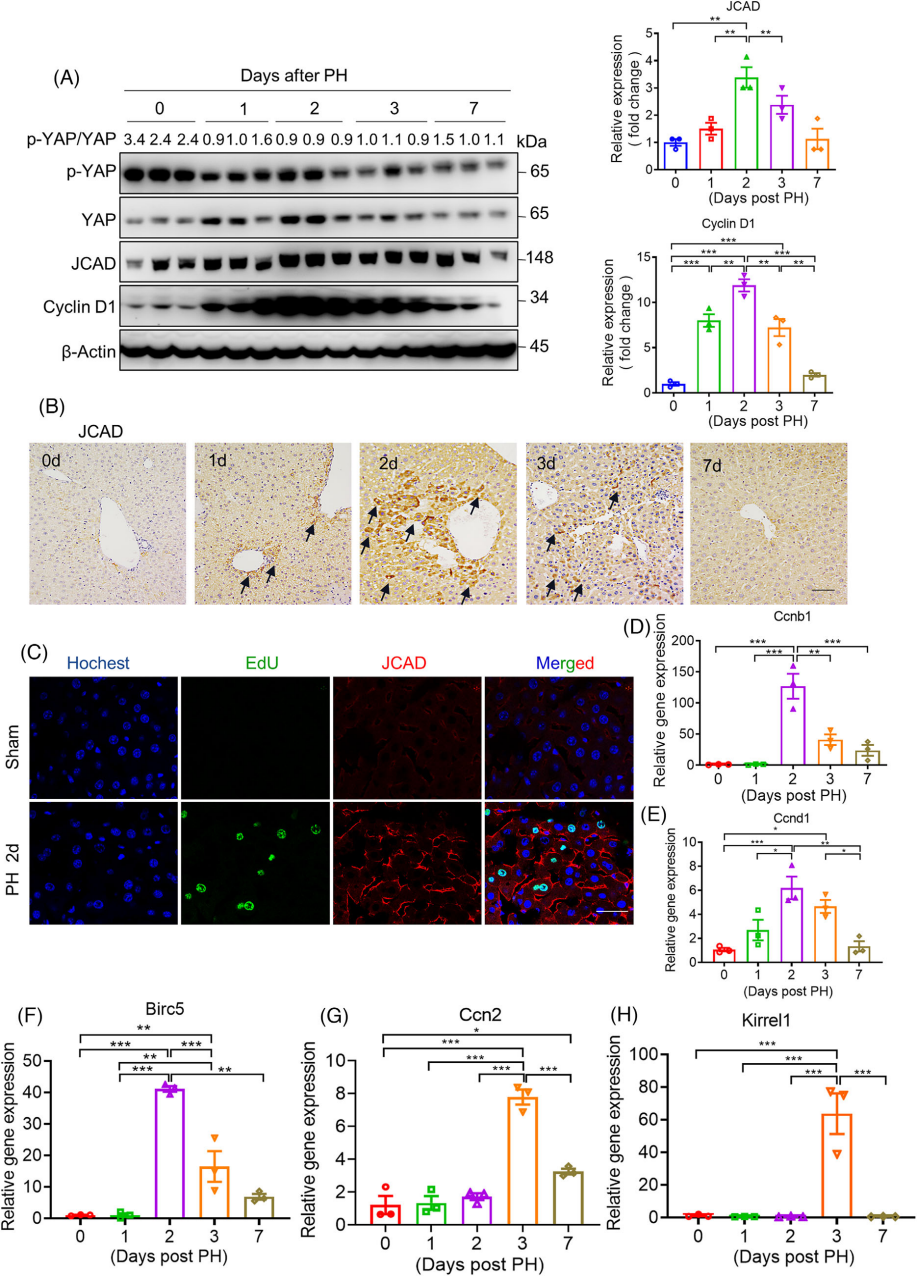
Junctional protein‐associated with coronary artery disease (JCAD) expression was increased in mice after partial hepatectomy (PH). Male wild‐type (WT) mice at 8‐week‐old were subjected to PH. (A) Western blotting was conducted to detect p‐YAP, Yes‐associated protein (YAP), JCAD and cyclin D1 levels in liver samples after 70% PH at different time points, and β‐actin was used as an internal control. Ratio of p‐YAP over YAP was presented as fold change of p‐YAP. Densitometric quantification of imaging bands for JCAD and cyclin D1 were shown (*n* = 3, one‐way analysis of variance [ANOVA] with Tukey's honest significant difference [HSD] test). (B) Representative immunohistochemical staining of JCAD at different time points after PH. The positive area was indicated by black arrow. Scale bars, 50 μm. (C) JCAD and 5‐ethynyl‐2′‐deoxyuridine (EdU) were co‐stained in liver samples 2 days post PH. Blue: nucleus; green: EdU; red: JCAD. Scale bars, 25 μm. (D–H) Relative gene expression of *Ccnb1* (cyclin B1), *Ccnd1* (cyclin D1), *Birc5*, *Ccn2* (CTGF) and *Kirrel1* was detected by quantitative reverse transcriptase polymerase chain reaction (RT‐qPCR) with β‐actin as the internal control (*n* = 3, one‐way ANOVA with Tukey's HSD test). All data are presented as mean ± standard error of mean (SEM), ^*^ *p* < .05, ^**^ *p* < .01, ^***^ *p* < .001 compared with control (Ctrl) group.

To investigate the potential involvement of JCAD in liver regeneration, C57BL/6J (wild type [WT]) mice were subjected to PH, which had been considered as a classic approach to investigate in vivo regenerative capacity. As shown in Figure 1A–C, JCAD‐positive cells presented in a portal area‐centred distribution, which is consistent with YAP localisation as previously reported.^22^ As a tight junction protein, expression of JCAD at the surface of hepatocyte was increased to a large extent post PH, which is in concordance with DNA biosynthesis when counterstained by 5‐ethynyl‐2′‐deoxyuridine (EdU). JCAD levels were increased quickly and peaked within 2 days post PH, and declined to a nearly base level from day 3 to 7 as evidenced by Western blot (WB) analysis and immunohistochemical staining, suggesting that JCAD participated in liver regeneration. Moreover, transcriptional expression of cyclin B1 and D1 exhibited a dynamic change in the pattern similar to JCAD post PH (Figure 1D,E). As an effector of the Hippo signalling, YAP protein content was increased during the 1st and 2nd days, accompanied with p‐YAP change in an opposite trend, resulting in a decrease in the p‐YAP/YAP ratio, which indicated that the Hippo–YAP signalling contributed to hepatic regeneration, in consistent with the dynamic change of YAP protein (Figure 1A). Consistently, the Hippo–YAP pathway downstream gene *Birc5* was markedly increased at the 2nd day (Figure 1F); whilst *Ccn2* and *Kirrel1* peaked at the 3rd day (Figure 1G,H). However, transcriptional level of *Yap1* (YAP) was slightly changed during PH, indicating that post‐transcriptional modulation of YAP rather than transcriptional change contributed to liver regeneration after PH (Figure S1A). Therefore, it was evident that regenerative hepatocytes exhibited a transient increase in JCAD expression after PH.

### 2.2 JCAD deficiency decelerated liver regeneration post PH

**FIGURE 2:**
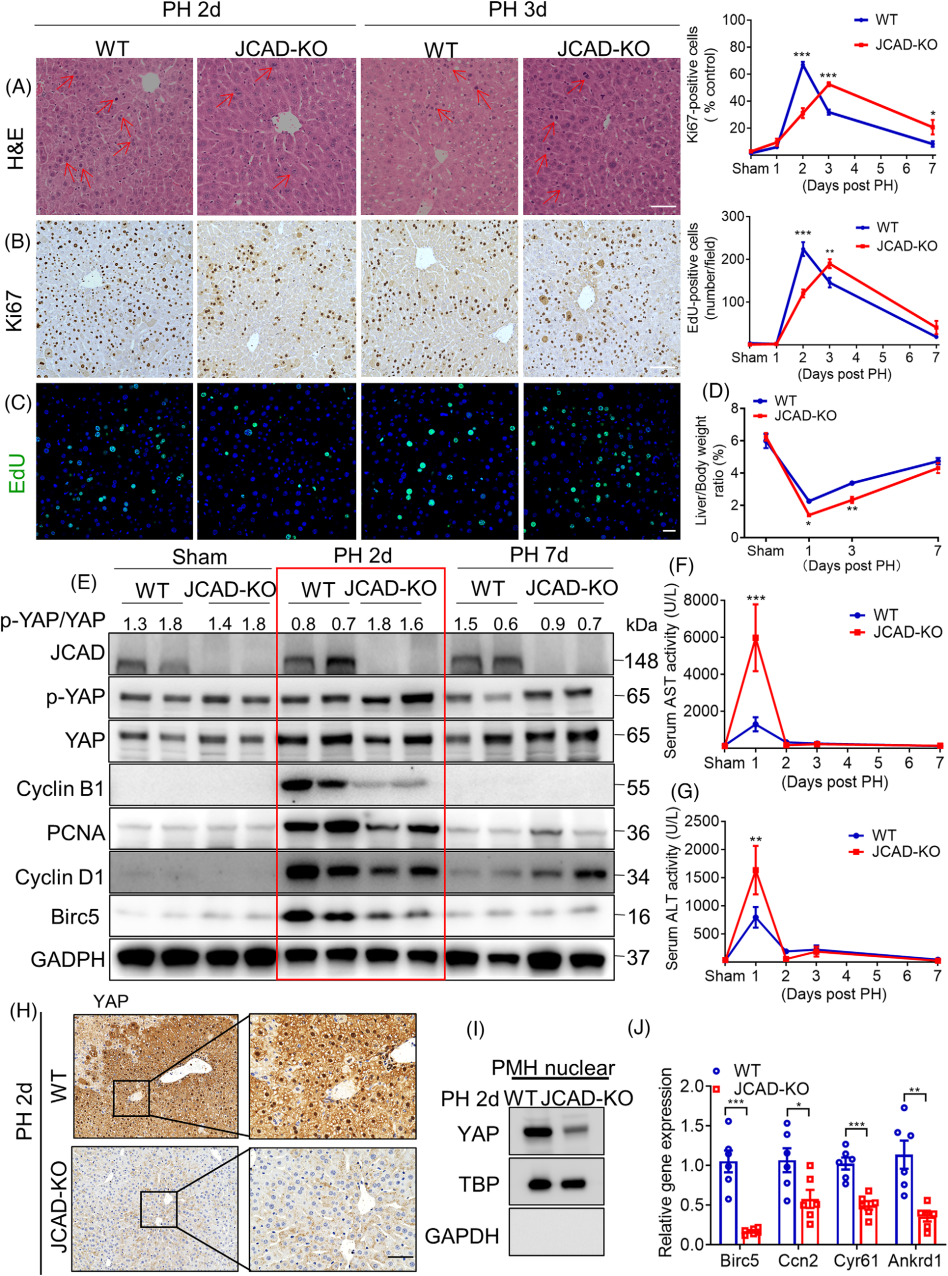
Junctional protein‐associated with coronary artery disease (JCAD) deficiency decelerated liver regeneration after partial hepatectomy (PH). (A–C) Representative micrographs of haematoxylin and eosin (H&E), Ki67 and 5‐ethynyl‐2′‐deoxyuridine (EdU) staining in wild‐type (WT) and JCAD knockout (JCAD‐KO) mice 2 or 3 days post PH (*n* = 6, two‐way analysis of variance [ANOVA] with Tukey's multiple comparisons). Mitotic hepatocytes were indicated by red arrows in the H&E‐stained liver sections. Ki67‐positive rate was presented as a ratio of positive cells/total cells in 100× filed. EdU‐positive cells were counted according to the fluorescent cells per 100× field. At least five fields were counted for each sample. Scale bars, 50 μm. (D) Ratio of liver over body weight was presented as ST/M (ST: harvested liver residue post PH; M, original mouse weight) (*n* = 6, two‐way ANOVA with Tukey's multiple comparisons). (E) Expression of proliferative cell nuclear antigen (PCNA), cyclin B1 and cyclin D1 in liver tissue of JCAD‐KO mice and WT mice 2 or 3 days post PH was determined by Western blotting (WB), and glyceraldehyde phosphate dehydrogenase (GAPDH) was used as a loading control. Serum aspartate aminotransferase (AST) (F) and alanine aminotransferase (ALT) (G) levels in JCAD‐KO and WT control post PH at indicated time points (*n* = 6, two‐way ANOVA with Tukey's multiple comparisons). (H) Immunohistochemical staining of Yes‐associated protein (YAP) in WT and JCAD‐KO mice 2 days post PH. Scale bars, 50 μm. (I) Nuclear expression of YAP in primary mouse hepatocytes (PMH) isolated in WT and JCAD‐KO mice 2 days post PH. (J) Relative expressions of YAP target genes in WT and JCAD‐KO mice 2 days post PH (*n* = 6, Student's *t*‐test). All data are presented as mean ± standard error of mean (SEM), ^*^ *p* < .05, ^**^ *p* < .01, ^***^ *p* < .001 compared to WT mice. TBP, TATA‐binding protein.

To further clarify the specific function of JCAD during liver regeneration, PH was conducted in both JCAD knockout (JCAD‐KO) and WT mice. As shown in Figure 2A–C, JCAD deficiency impeded hepatocellular proliferation as evidenced by mitotic hepatocytes (double nuclei), Ki67 staining and in situ EdU incorporation compared to WT mice, and the proliferative peak was postponed to the 3rd day (Figure 2B,C). Consistently, JCAD‐KO mice exhibited a lower ratio of liver over body weight than WT mice after PH (Figure 2D), whilst comparable liver regeneration index (Figure S1B), indicating an impeded recovery of liver mass after PH in comparison to WT mice. Expression levels of critical cell cycle‐associated proteins, such as cyclin D1, cyclin B1 and proliferating cell nuclear antigen (PCNA) in JCAD‐KO mice were significantly lower than WT mice at the proliferative peak 2 days post PH (Figures 2E and S1C–G). Moreover, JCAD deficiency caused a transient elevation in serum alanine aminotransferase (ALT) and aspartate aminotransferase (AST) levels, suggesting JCAD‐KO mice exhibited more severe hepatocellular injury than WT mice (Figure 2F,G). In consistent with the reduced YAP activity shown in Figure 2E, YAP nuclear localisation and expression of YAP target genes were blunted in JCAD‐KO mice 2 days after PH (Figure 2H–J). To conclude, these data demonstrated that the deletion of JCAD resulted in impaired liver regeneration and a delayed regenerative peak after PH in mice, which is associated with the Hippo–YAP signalling activity.

### 2.3 Hepatocyte‐specific deletion of JCAD impaired liver regeneration post PH

**FIGURE 3:**
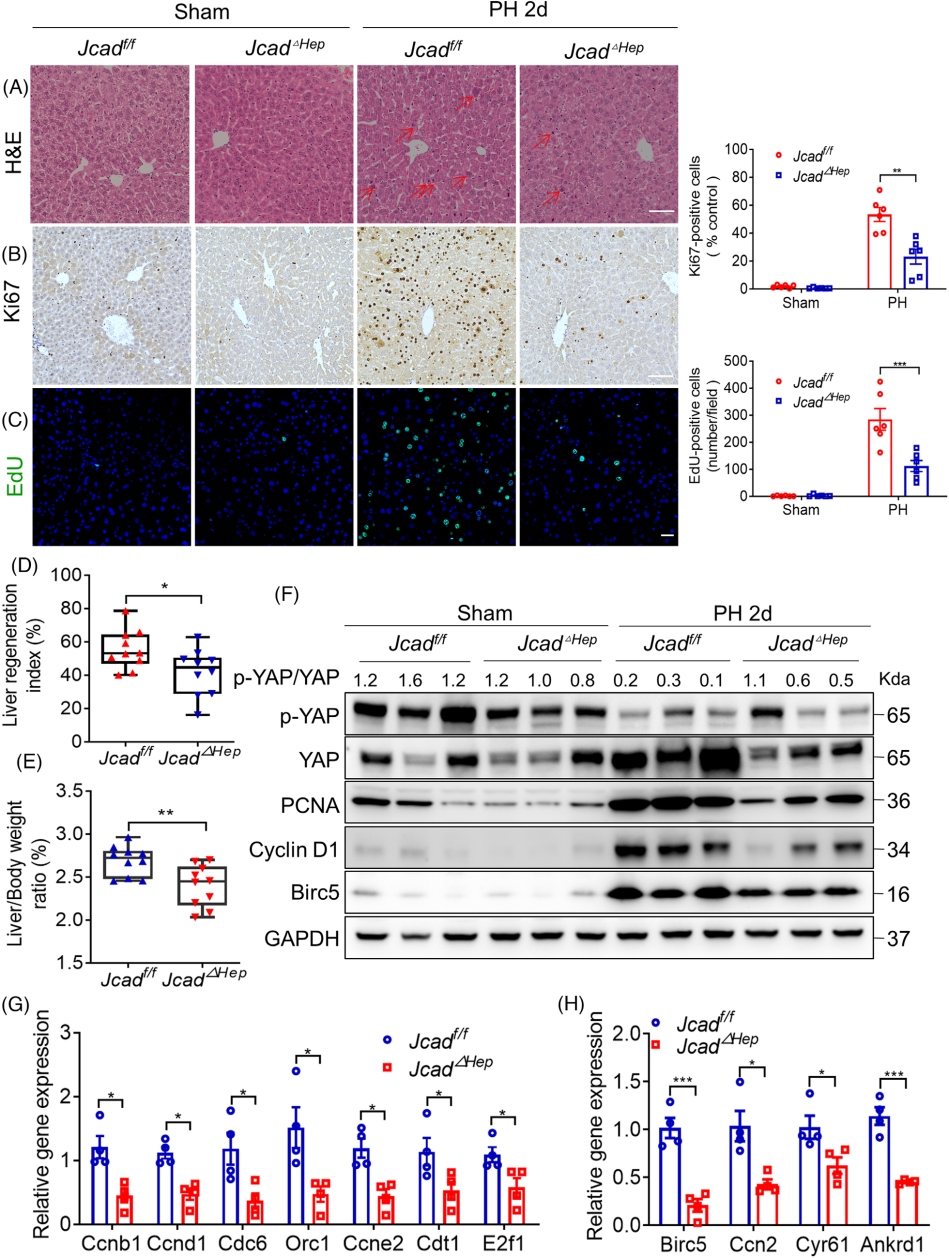
Hepatic junctional protein‐associated with coronary artery disease (JCAD) deficiency decelerated liver regeneration after partial hepatectomy (PH). (A–C) Representative micrographs of haematoxylin and eosin (H&E), Ki67 and 5‐ethynyl‐2′‐deoxyuridine (EdU) staining in liver sections of *Jcad^f/f^* and *Jcad^△Hep^* mice 2 days post PH. Mitotic hepatocytes were indicated by arrows in the H&E‐stained liver sections. Ki67 and EdU‐positive cells were counted in 100× fields. Scale bars, 50 μm (*n* = 6, two‐way analysis of variance [ANOVA] with Tukey's multiple comparisons). (D and E) Index of liver regeneration and ratio of liver over body weight were reduced in *Jcad^△Hep^* mice compared to *JCAD^f/f^* post PH (*n* = 10, Student's *t*‐test). (F) Expression of proliferation‐related proteins in *Jcad^f/f^* and *Jcad^△Hep^* mice was measured by Western blotting (WB), and GAPDH was used as a loading control. (G) Hepatic expression of key genes involved in cell cycle, such as *Ccnb1* and *Ccnd1*, was suppressed in *Jcad^△Hep^* mice (*n* = 4, Student's *t*‐test). (H) Relative expressions of Yes‐associated protein (YAP) target genes in *JCAD^f/f^* and *Jcad^△Hep^* mice 2 days post PH (*n* = 4, Student's *t*‐test). All data are presented as mean ± standard error of mean (SEM). ^*^ *p* < .05, ^**^ *p* < .01, ^***^ *p* < .001 compared to *JCAD^f/f^* mice.

Given that an increase in hepatocyte number and cell volume is the major factor contributing to liver regeneration post PH,^23^ hepatocyte‐specific JCAD‐KO (denoted as *Jcad^△Hep^* in short) mice were generated by crossing *Jcad^f/f^* mice by floxing the JCAD‐coding region in the exon 3 with albumin‐cre mice (Figure S2A). *Jcad^△Hep^* mice and their WT (*Jcad^f/f^*) littermates were subjected to PH, and sacrificed 2 days after PH. Liver‐specific JCAD deficiency manifested with impeded regenerative activity as demonstrated by diminished hepatocellular mitosis, Ki67‐positive cells and EdU incorporation (Figure 3A–C). Although ALT and AST levels were comparable (Figure S2B,C), liver mass recovery was impaired in *Jcad^△Hep^* mice as shown by the decrease in a ratio of liver over body weight and liver regenerative index in *Jcad^△Hep^* mice compared to *Jcad^f/f^* mice (Figure 3D,E). In consistency with the result in JCAD‐KO mice, immunoblotting and quantitative reverse transcriptase polymerase chain reaction (qRT‐PCR) analyses showed that JCAD deficiency in hepatocytes caused a profound reduction in transcription of cell cycle‐associated genes, such as cyclin D1, cyclin B1 and cyclin E2 (Figures 3F,G and S2D–G). Moreover, YAP protein level was significantly decreased, whilst p‐YAP/YAP ratio was increased in *Jcad^△Hep^* mice compared to *Jcad^f/f^* mice, indicating impaired YAP signalling activity, which was also validated by decreased YAP target gene expression (Figure 3F–H). To sum up, these data revealed that JCAD deficiency in the liver resulted in deceleration of regeneration post PH in a YAP‐dependent manner.

### 2.4 Cell cycle arrest in the liver of JCAD‐KO mice post PH

**FIGURE 4:**
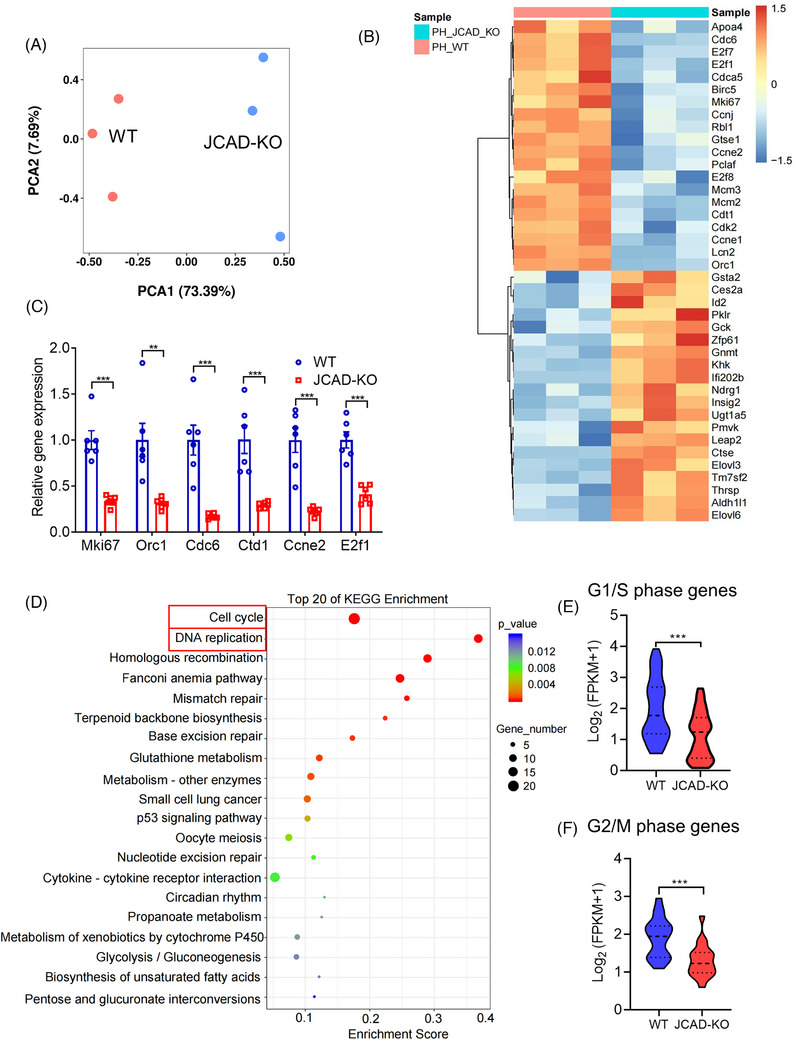
RNA‐sequencing analysis of liver samples in wild‐type (WT) and junctional protein‐associated with coronary artery disease knockout (JCAD‐KO) mice 2 days post partial hepatectomy (PH). (A) Principal component analysis (PCA) was performed in RNA‐sequencing data of WT and JCAD‐KO mice 2 days post PH. (B) Heatmap of gene expression profiles post PH. (C) mRNA levels of genes involved in cell cycle were determined by reverse transcriptase polymerase chain reaction (RT‐PCR), and presented as relative expression levels using β‐actin as a house‐keeping gene control (*n* = 6, Student's *t*‐test). (D) Kyoto Encyclopedia of Genes and Genomes (KEGG) pathway analysis demonstrated that the terms associated with the cell cycle and DNA replication are the most enriched in the differential genes. (E and F) Cell cycle genes characteristic of the S phase (*n* = 51, Student's *t*‐test) and G2‐M transition (*n* = 18, Student's *t*‐test) were significantly downregulated by JCAD‐KO, the median (middle line), 25th and 75th percentile (dot plot) was indicated. All data are presented as mean ± standard error of mean (SEM). ^**^ *p* < .01, ^***^ *p* < .001 compared to WT mice.

To investigate underlying mechanisms of the suppressed liver proliferative capability of JCAD‐KO mice, RNA sequencing (RNA‐Seq) analysis was undertaken in liver tissue of JCAD‐KO and WT mice 2 days post PH, given the fact that this time point represented as the peak in regenerative response, as shown in Figure 1. Almost 74% of the variance between JCAD‐KO and WT groups could be explained by the first principal component, and the three samples collected under each condition clustered closely along the axis in the principal component analysis (Figure 4A). It is evident that JCAD deficiency caused down‐regulation in a number of genes involved in the cell cycle machinery, such as *Ki67*, *Ccne2*, *Cdk2*, *Cdt1*, *E2f1*, etc., suggesting a transition from G1 to S and mitotic block occurred in JCAD‐KO mice (Figure 4B). Furthermore, mRNA levels of major differential genes were validated by RT‐PCR (Figure 4C). Kyoto Encyclopedia of Genes and Genomes (KEGG) pathway enrichment was conducted to clarify underlying pathways contributing to this phenomenon. Notably, genes associated with cell cycle and DNA replication are predominant in enriched datasets, suggesting that cell cycle and DNA replication were retarded in regenerative response in JCAD‐KO mice after PH (Figure 4D). To assess the changes in gene expression characterised by cell cycle transition triggered by JCAD‐KO, fragments per kilobase per million values for cell cycle (KEGG term 04110), which stand for the genes enriched in this KEGG term and the genes specific to the S and G2/M stages, were remarkably down‐regulated in JCAD‐KO mice, whilst apoptosis‐related genes remained unchanged, indicating that JCAD deficiency arrested cell cycle transition; however, they had less significant impact on apoptosis‐related events during liver regeneration, as validated by WB analysis and flow cytometry of propidium iodide/Annexin V in JCAD‐KO and siRNA‐transfected cell line (Figures 4E,F and S3). These results indicated that a global JCAD eradication resulted in a suppressed capacity to enter the cell cycle, and retarded proliferation in restoration of the original liver mass post PH.

### 2.5 JCAD sensitised hepatocytes to epidermal growth factor stimulation via Hippo–YAP signalling pathway

**FIGURE 5:**
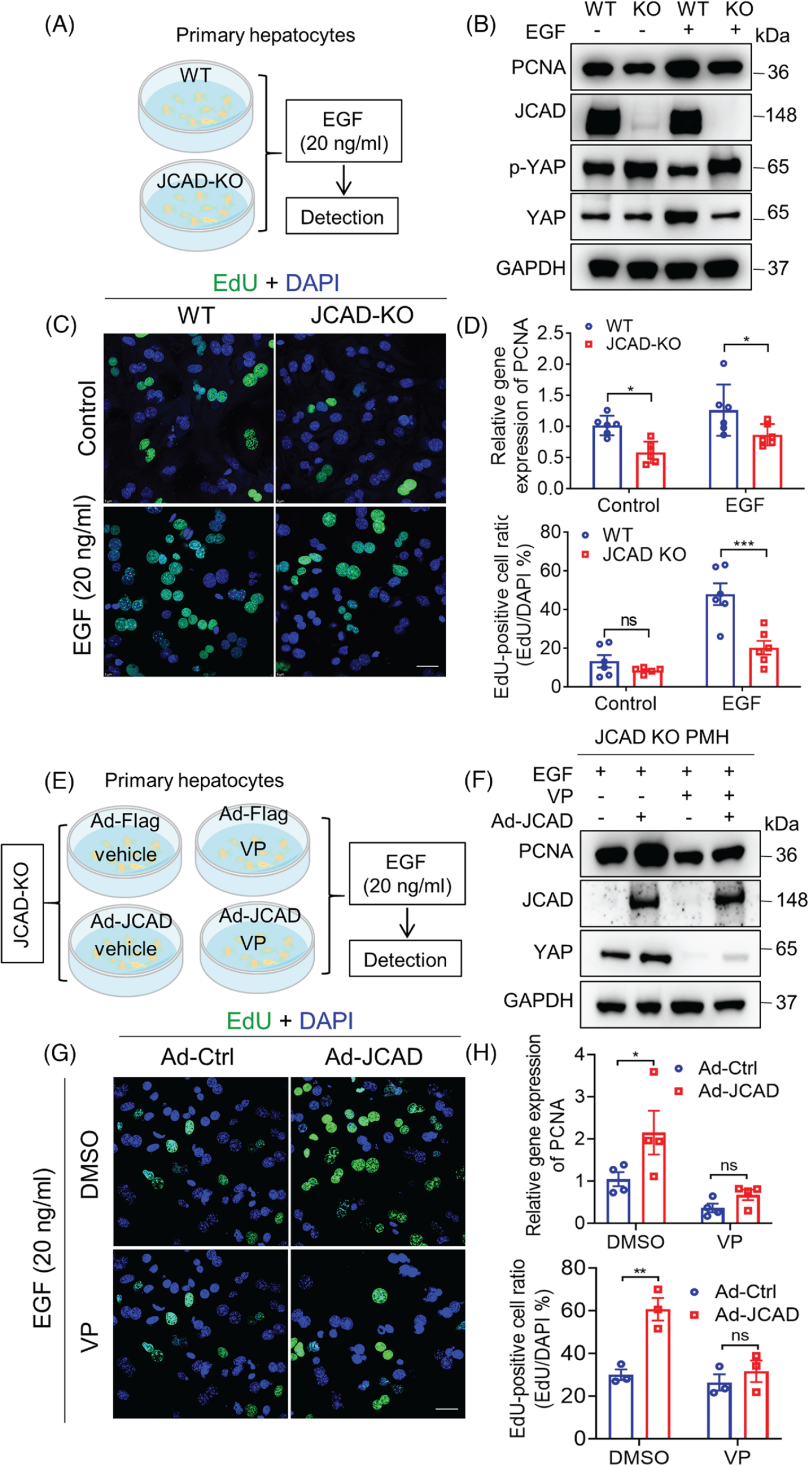
Junctional protein‐associated with coronary artery disease (JCAD) sensitised primary hepatocytes to epidermal growth factor (EGF) stimulation via Hippo signalling pathway. (A) Primary hepatocytes were isolated from wild‐type (WT) and JCAD knockout (JCAD‐KO) mice and stimulated with 20 ng/mL murine EGF for 24 h. (B) Proliferating cell nuclear antigen (PCNA), p‐YAP and Yes‐associated protein (YAP) protein levels were determined by Western blot analysis. (C) Less 5‐ethynyl‐2′‐deoxyuridine (EdU)‐positive cells were stained with or without EGF stimulation. Ratio of EdU‐positive cells was presented as EdU‐positive cells over 4′,6‐diamidino‐2‐phenylindole (DAPI) per field (*n* = 6, two‐way analysis of variance [ANOVA] with Tukey's multiple comparisons). At least five fields were counted. (D) PCNA gene expression was less responding in JCAD‐KO hepatocytes with or without EGF (*n* = 6, two‐way ANOVA with Tukey's multiple comparisons). (E) The cell‐processing flow diagram is described as follows. Primary hepatocytes were isolated from JCAD‐KO mice and infected with either control adenovirus (Ad‐Ctrl) or JCAD expressing adenovirus (Ad‐JCAD) for 48 h, at 24 h post infection, 20 ng/mL EGF with or without verteporfin (VP) (5 μM) was added for another 24 h. (F–H) VP abolished increased expression of PCNA and EdU incorporation induced by Ad‐JCAD infection (H, *n* = 4; G, *n* = 3, two‐way ANOVA with Tukey's multiple comparisons). At least five images were taken for each treatment. Scale bars, 25 μm. Representative images were chosen from three independent experiments. All data are presented as mean ± standard error of mean (SEM), ^*^ *p* < .05, ^**^ *p* < .01, ^***^ *p* < .001 compared to control group.

In order to investigate the efficiency of hepatocellular proliferative response to stimulation with epidermal growth factor (EGF), primary hepatocytes were isolated from JCAD‐KO or WT mice (Figure 5A). The treatment with EGF at 20 ng/mL for 24 h strikingly triggered PCNA expression in both WT and KO hepatocytes; however, there was a trend towards lower PCNA levels in JCAD‐KO hepatocytes than WT group (Figure 5B,D). The ratio of p‐YAP over YAP in EGF‐treated hepatocytes displayed in a similar trend between JCAD‐KO mice and WT mice after PH, which pointed to that YAP activity was impaired in JCAD‐KO hepatocytes (Figure 5B). In consistent with PH data, the magnitude of elevation in EdU‐positive ratio was more pronounced in hepatocytes from WT mice than those from JCAD‐KO mice (Figure 5C). Furthermore, adenovirus‐mediated overexpression of JCAD in WT primary hepatocytes enhanced DNA replication as indicated by increased PCNA expression and EdU incorporation as well as activated YAP activity (Figure S4A–C). To examine whether the enhanced proliferative effect of JCAD under EGF treatment was dependent on YAP activation, primary hepatocytes of JCAD‐KO mice were treated with a YAP inhibitor, verteporfin (VP), followed by EGF stimulation. Remarkably, replenishment of JCAD in primary hepatocytes from JCAD‐KO mice restored the impaired DNA replication, whilst the proliferative effects of JCAD were largely abolished by addition of VP (Figure 5E–H). These results demonstrated that JCAD sensitised hepatocytes to EGF stimulation through the Hippo–YAP signalling pathway.

### 2.6 JCAD deficiency resulted in mitotic blockage

**FIGURE 6:**
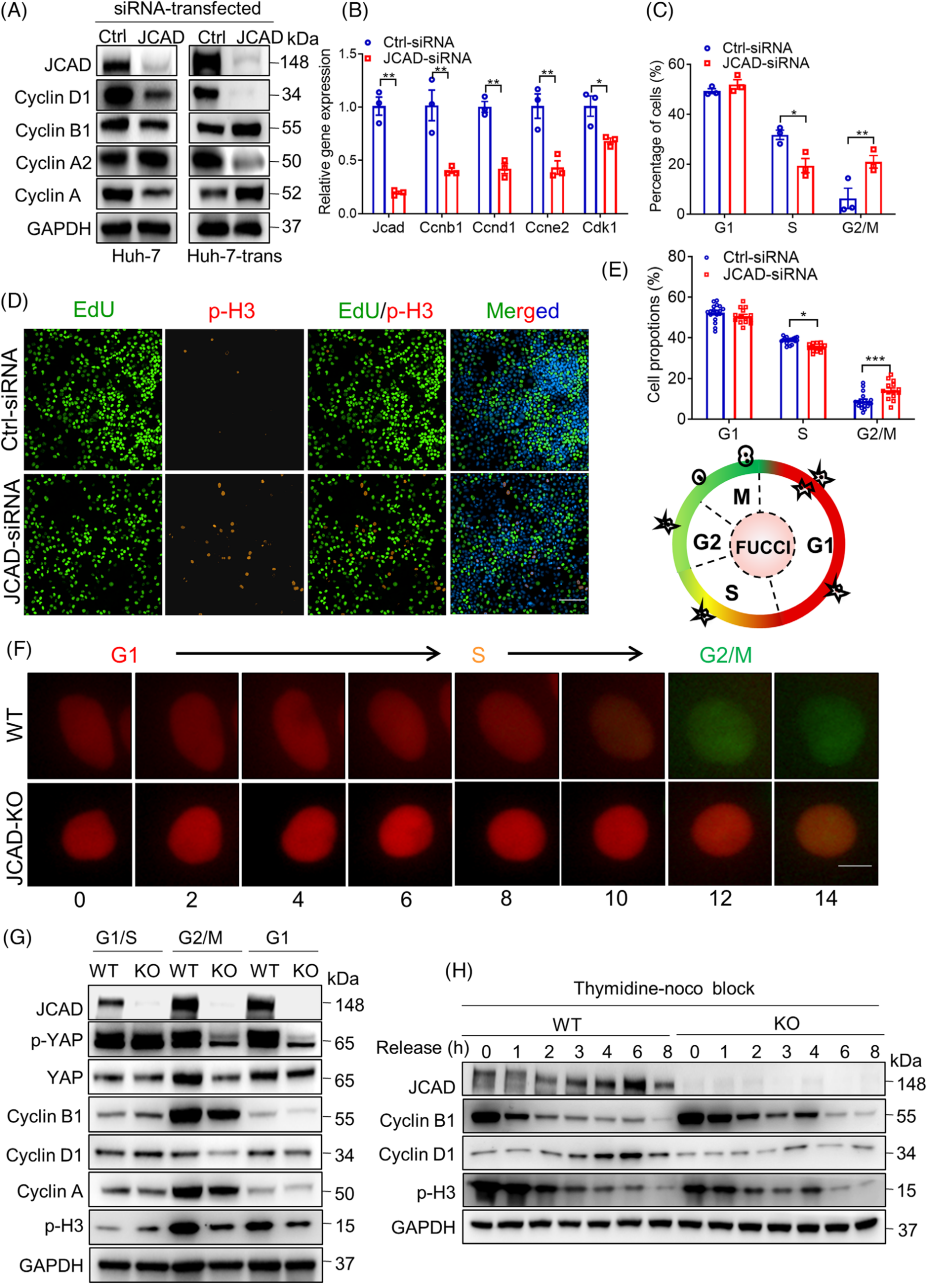
Junctional protein‐associated with coronary artery disease (JCAD) deficiency resulted in mitosis blockage. (A) Western blot of cell cycle‐associated proteins in cell lines transfected with JCAD‐siRNA. (B) Expression of genes associated with cell cycle checkpoint of Huh‐7 cells transfected with JCAD‐siRNA (*n* = 3, Student's *t*‐test). (C) Distribution of cell cycle phases was measured by flow cytometry followed by propidium iodide (PI) staining (*n* = 3, two‐way analysis of variance [ANOVA] with Tukey's multiple comparisons). (D) Representative images of 5‐ethynyl‐2′‐deoxyuridine (EdU) and phosphohistone 3 (p‐H3) staining. Scale bars, 100 μm. (E) Phase proportion of cell cycle was depicted by p‐H3 (G2/M) and EdU (S) staining after siRNA transfection (*n* = 14, two‐way ANOVA with Tukey's multiple comparisons). (F) Live imaging of cell cycle phases in a high content screening modality for JCAD knockout (JCAD‐KO) Huh‐7 cells transfected with a fluorescent ubiquitination‐based cell cycle indicator (FUCCI) system. Cell cycle transition was impeded in JCAD‐KO cells, and images were taken every 2 h for total of 24 h (data not shown for shots taken 16–24 h for next round of cell cycle entrance in wild‐type [WT] group, red: G1 phase, yellow: S phase, green: G2/M phase). Scale bar, 10 μm. (G) Entrance into G2/M phase of JCAD‐KO Huh‐7 cells was postponed after being released from S phase synchronisation by double thymidine block. (H) JCAD‐KO cells exhibited prolonged expression of cyclin B1 and p‐H3, and cyclin D1 failed to increase from the basic level after released from G2/M phase synchronisation by thymidine–nocodazole block. Ccnb1: cyclin B1; Ccnd1: cyclin D1; Ccne1: cyclin E1; Cdk1: cyclin‐dependent kinase‐1. Every experiment was repeated for three times. All data are presented as mean ± standard error of mean (SEM), compared to control (Ctrl) group, ^*^ *p* < .05, ^**^ *p* < .01.

To further investigate how JCAD affects the cell cycle machinery, three pairs of siRNAs against JCAD were transfected into Huh‐7 cells to examine the knockdown efficiency, and all of them exhibited a desirable efficacy (Figure S5A,B), amongst them JCAD‐siRNA3 was randomly selected for subsequent investigation. In accordance with the in vivo results that JCAD deficiency decelerated liver regeneration, Huh‐7 and Huh‐7‐trans cells were applied in this study since JCAD expression is apparent in these two cell lines.^24^ JCAD knockdown by siRNA in Huh‐7 and Huh‐7‐trans cells, the latter is a high‐metastatic hepatoma cells derived from Huh‐7 cells, resulted in down‐regulation of cyclin D1 at both mRNA and protein levels (Figure 6A,B). To investigate which checkpoint was affected, flow cytometric analysis of cell cycle transition was conducted in Huh‐7 cells. As shown in Figures 6C and S5C, JCAD knockdown led to a higher proportion of G2/M phase distribution in accompany with less S phase proportion than cells transfected with control siRNAs, which indicated that JCAD inhibition might give rise to a mitotic block. To further validate this hypothesis, EdU and phosphohistone 3 (p‐H3) staining in live cells was conducted, and visualised in a high‐content live‐imaging modality. p‐H3 is well‐recognised as a characteristic marker for G2/M phase, which exists at later G2 phase and increases at M phase, while EdU is a DNA synthesis marker, and represents S phase activity. As shown in Figure 6D,E, EdU staining was reduced, and in contrast p‐H3 signal was significantly increased in JCAD‐deficient cells compared to those transfected with control siRNA. These data demonstrated that JCAD suppression blocked cell cycle entrance into the S phase and resulted in mitotic blockage.

JCAD was knocked‐out by a CRISPR‐Cas9 approach in Huh‐7 and Huh‐7‐trans cells to further investigate its role in cell cycle transition (Figure S5D). Stable JCAD‐KO clones of Huh‐7 cells were further seeded in a single cell base (Figure S5E). In consistent with cells with down‐regulation of JCAD, expression of proteins associated with cell cycle transition, such as cyclin B1, cyclin D1, cyclin A and cyclin A2, was suppressed in JCAD‐KO Huh‐7 cells in comparison to the controls (Figure S5F). To visualise cell cycle phases in a real‐time fashion upon JCAD‐KO, a fluorescent ubiquitination‐based cell cycle indicator (FUCCI) system was transfected into Huh‐7 cells. This powerful tool is based on the phase‐dependent proteolysis of the oscillators Cdt1 and geminin. Fusion protein of Cdt1 with the mAG (hCdt1‐mAG) serves as indicators of G1, whilst geminin with the mKO2 (hGeminin‐mKO2) represents S and G2/M phases, respectively.^18^ Control cells transfected with FUCCI presented a complete transition from G1 to G2/M phases within 14 h on average; in contrast, cell cycle was delayed from G1 to S phase for more than 14 h in JCAD‐KO cells (Figure 6F). Presumably, it appeared that JCAD deficiency led to cell cycle arrest, and altered phase distribution in cell cycle further supported this speculation (Figure 6G,H). Double thymidine block (DTB) and thymidine‐noco blocking assays were further conducted to examine expression of downstream cell cycle‐associated proteins in different phases. As reported previously, cell cycle transition was completed within 16 h in Huh‐7 cell as validated by flow cytometry (Figure S6A), and cyclin B1 accumulation was peaked at the G2/M phase in accordance with p‐H3, whilst cyclin D1 expression was increased during the transition from G1 to S phase (Figure S6B,C). JCAD‐KO and control cells were synchronised into a G1/S phase by DTB and released to examine downstream protein variation for 16 h. As shown in Figure 6G, expression of cyclin B1 and p‐H3 was reduced in JCAD‐KO cells, indicating that cell cycle entrance from S phase to G2/M phase was blocked. Furthermore, when they were synchronised to the G1 phase by thymidine‐noco blockage, cyclin B1 and p‐H3 exhibited extended expression after release, whilst cyclin D1 failed to increase from a basal level upon JCAD deficiency, suggesting a prolonged retention of G1 phase and failure to transit at the G1/S checkpoint in JCAD‐KO cells (Figure 6H). In consistent with siRNA transfection, JCAD‐KO in Huh‐7‐trans cell line led to a higher proportion of cells in a G2/M phase (Figure S7A,B) and a lower S phase distribution as presented by EdU flow cytometry than controls (Figure S7C), which further validated that the lack of JCAD postponed mitosis in Huh‐7‐trans cells. Moreover, JCAD‐KO significantly impeded cell proliferation as evidenced by methyl thiazolyl tetrazolium assay (Figure S7D,E). Taken together, these data demonstrated that JCAD‐KO blocked cell cycle transition with retardation in cell proliferation.

### 2.7 JCAD modulated cell cycle transition in a Hippo–YAP pathway‐dependent manner

**FIGURE 7:**
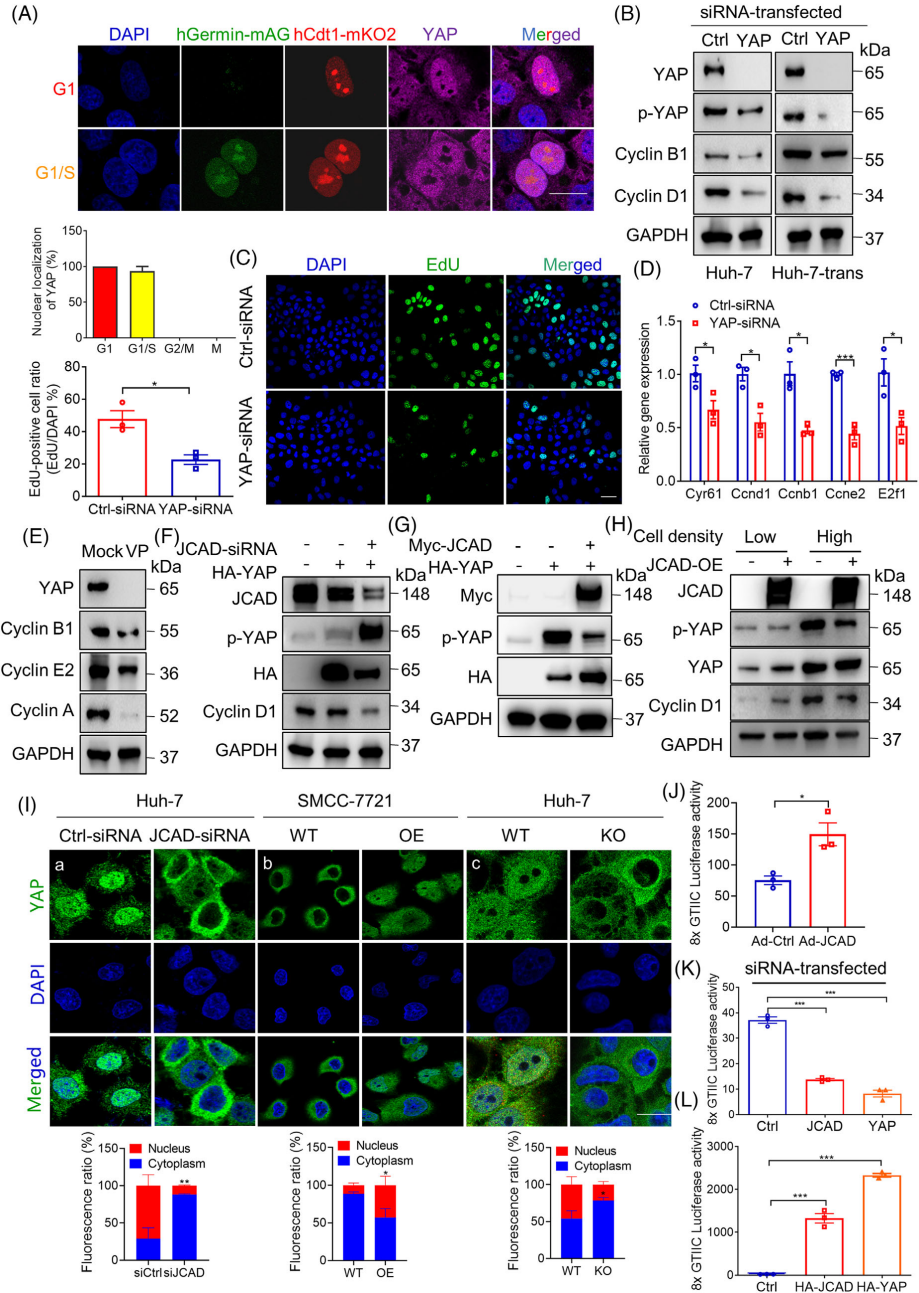
Junctional protein‐associated with coronary artery disease (JCAD) modulate cell cycle transition in a Hippo–YAP pathway‐dependent manner. (A) Yes‐associated protein (YAP) nuclear localisation was mainly distributed in G1 and S phase. Huh‐7 cells transfected with fluorescent ubiquitination‐based cell cycle indicator (FUCCI) were stained with YAP, and YAP nuclear localisation were visualised in different phases of cell cycle by FUCCI live imaging. Scale bars, 20 μm. (B) Western blot analysis of proteins involved in cell cycle in Huh‐7 and Huh‐7‐trans cells transfected with control or YAP‐siRNA. (C) Cell transfected with YAP‐siRNA exhibited reduced 5‐ethynyl‐2′‐deoxyuridine (EdU) staining. EdU‐positive cell ratio was presented by EdU‐positive cells/4′,6‐diamidino‐2‐phenylindole (DAPI) per field (*n* = 3, Student's *t*‐test). Scale bars, 50 μm. (D) Gene expression of cell cycle checkpoint‐related proteins in Huh‐7 cells transfected with YAP‐siRNA (*n* = 3, Student's *t*‐test). (E) YAP inhibitor verteporfin (VP) reduced expression of cell cycle‐related proteins. Huh‐7 cells were treated with 5 μM VP for 24 h. (F) Huh‐7 cells were transfected with JCAD‐siRNA or HA‐YAP, and expression of Hippo signal pathway proteins was determined. (G) HA‐YAP were transfected with or without Myc‐JCAD to Huh‐7 to modulate p‐YAP activity. (H) JCAD‐OE cells were plated at different cell density, and key proteins in the Hippo signal pathway were determined by Western blot analysis. (I) YAP nuclear and cytosol distribution was determined in cells transfected with JCAD‐siRNA (a) or stably overexpressing JCAD (b) or with JCAD stably knocked‐out (c). At least five fields for each treatment were counted. Scale bars, 20 μm. (J–L) JCAD enhanced YAP transcriptional activity. Wild‐type (WT) mouse primary hepatocyte infected with JCAD expressing adenovirus (Ad‐JCAD) (J) and Huh‐7 cells were transiently transfected with transcriptional enhanced associate domain (TEAD)‐responsive 8xGTIIC luciferase reporter gene (Renilla luciferase reporter plasmid pRL‐SV40 as an internal control) together with JCAD‐siRNA (K) or HA‐JCAD plasmid (L), and YAP‐siRNA or HA‐YAP was transfected as a positive control (*n* = 3, Student's *t*‐test in (J) and one‐way analysis of variance [ANOVA] with Tukey's HSD in (K) and (L)). Each experiment was repeated for three times. All data are presented as mean ± standard error of mean (SEM), compared with control (Ctrl) group, ^*^ *p* < .05, ^**^ *p* < .01, ^***^ *p* < .001.

To further examine the impact of the Hippo–YAP pathway on cell cycle transition, YAP cellular localisation was identified in cell cycle phase, and YAP was knocked‐down to examine cellular response. As shown in Figure 7A, YAP was counterstained in Huh‐7 cells transfected with FUCCI to clarify phase‐dependent YAP nuclear localisation, and it appeared that YAP was translocated into the nucleus mainly in G1 and S phases, indicating that YAP dephosphorylation may contribute to cell cycle transition.^18^ Moreover, three pairs of *YAP1* siRNAs were transfected to Huh‐7 cells to validate their knockdown efficiency, whilst siRNA2 and siRNA3 exhibited efficient down‐regulation of YAP, and siRNA3 was selected for further investigation (Figure S8A,B). In consistent with a previous report,^25^ *YAP1* knockdown by siRNA reduced levels of proteins involved in cell cycle at both mRNA and protein levels (Figures 7B,D and S8C). Furthermore, YAP1 siRNA transfection significantly reduced EdU‐positive cell count, indicating that cell cycle transition was repressed (Figure 7C). When cells were treated with VP, a well‐recognised YAP inhibitor (Figure S8D), similar results were obtained (Figure 7E). Taken together, these data indicate that YAP activity oscillated during cell cycle and might participate in regulating cell cycle transition.

Next, mechanisms underlying JCAD deficiency‐mediated cell cycle transition blockage were investigated. Given the crucial function of the Hippo–YAP signalling pathway in initiation and termination of regenerative response, dual luciferase reporter assay, immunoblotting analysis and YAP nuclear trans‐localisation were employed to determine the effects of loss‐of‐function or gain‐of‐function of JCAD. JCAD loss‐of‐function by siRNA or sgRNA markedly enhanced p‐YAP activity (Figures 7F and S5F), whilst overexpression of JCAD reduced the phosphorylation of YAP activity (Figure 7G,H). Given that cell density may regulate the Hippo signalling activity, JCAD‐stably‐expressing cells were plated with a low and high density to investigate function of JCAD under different cell density. It turned out that under a high density, JCAD exhibited a notable efficacy (Figure 7H), possibly due to the fact that the Hippo signalling pathway was activated in high cell density. YAP nuclear and cytosol distribution was further determined in cells transfected with JCAD knockdown (siRNA), overexpression (stably expressing JCAD) or knockout (CRISPR/cas9 for JCAD). It was found that JCAD deficiency sequestered YAP in the cytosol, whilst overexpression of JCAD resulted in YAP nuclear trans‐localisation, suggesting that YAP activity was induced by JCAD (Figure 7I). Moreover, 8xGTIIC luciferase reporter activity was significantly increased by JCAD overexpression, whilst JCAD deficiency resulted in an opposite trend (Figure 7J–L), suggesting that JCAD functions as a positive regulator of YAP transcriptional activity. In summary, these data indicate that JCAD participated in the modulation of cell cycle transition in a YAP‐dependent manner.

### 2.8 JCAD modulated the Hippo signalling pathway through interacting with WWC1

**FIGURE 8:**
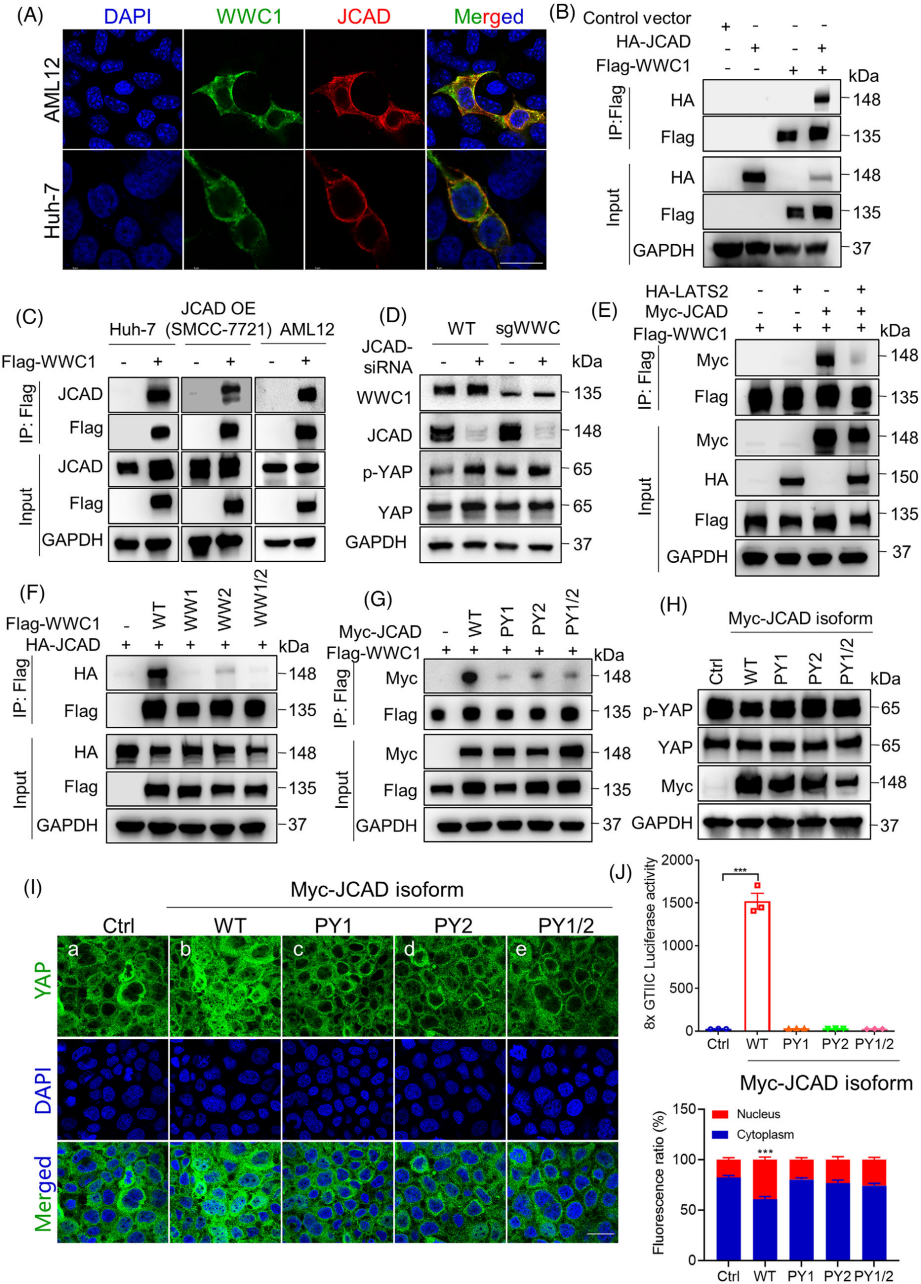
Junctional protein‐associated with coronary artery disease (JCAD) modulated the Hippo signalling pathway through interacting with WWC1. (A) JCAD and WWC1 were co‐localised in cytoplasmic membrane and proximity. AML12 and Huh‐7 cells were transfected with Flag‐WWC1 and HA‐JCAD; Flag‐tag and HA‐tag were stained in green and red, respectively. Scale bar, 20 μm. (B) HEK293T cells were transiently transfected with HA‐JCAD and Flag‐WWC1. Co‐immunoprecipitation (Co‐IP) analysis was performed. (C) Flag‐WWC1 was transfected in Huh‐7 (which highly expressed JCAD), SMCC‐7721 cells with JCAD stably overexpression as well as AML12 cells, and Flag‐tag was immunoprecipitated followed by JCAD immunoblotting. (D) HEK293A cells with WWC stably knocked‐down were transiently transfected with JCAD‐siRNA; and p‐YAP was determined by Western blot analysis. (E) Large tumour suppressor 2 (LATS2) competed with JCAD for binding to WWC1. HEK293T cells were transfected with Myc‐JCAD, HA‐LATS2 and Flag‐WWC1, followed by Co‐IP assay. (F) WWC1 interacted with JCAD at the WW domain in WWC1. WWC1 cells with mutations at WW1, WW2 or WW1/2 domains were transfected together with HA‐JCAD; and Co‐IP assay was conducted against Flag‐tag. (G) WWC1 interacted with JCAD at the PPxY domain in JCAD. PY1, PY2 or PY1/2‐mutated Myc‐JCAD were co‐transfected with Flag‐WWC1; and Co‐IP assay was conducted against Flag‐tag. (H–J) Huh‐7 cell was transfected with either wild type (WT) (b), PY1 (c), PY2 (d) or PY1/2 (e) mutant of Myc‐JCAD, and immunoblot of Yes‐associated protein (YAP) and p‐YAP (H) immunofluorescent assay of YAP (I) and 8xGTIIC dual luciferase assay (J) were conducted (*n* = 3, one‐way analysis of variance [ANOVA] with Tukey's HSD). Scale bars, 20 μm. All data are presented as mean ± standard error of mean (SEM), compared to control (Ctrl) group, ^***^ *p* < .001.

JCAD harbours two PPxY motifs, a short peptide sequence that is recognised by WW domains.^20^ The PPxY and WW domain‐mediated protein–protein interactions are widely present amongst Hippo signalling components especially in WWC family proteins, which harbour two WW domains interacting with PPxY with high affinity, indicating that JCAD may exert its function by interacting with WWC.^21^ WWC family proteins have recently been shown to interact with both LATS1/2 and MST1/2–SAV1, and mediate LATS1/2 activation by MST1/2.^10^ It was found that JCAD and WWC1 were co‐localised in cytoplasmic membrane in the present study, suggesting that these two proteins may physically interact with each other (Figure 8A). Indeed, both co‐immunoprecipitation (Co‐IP) assays for endogenous and ectopically expressed proteins supported a molecular interaction between JCAD and WWC1 (Figure 8B,C). Moreover, JCAD knockdown by siRNA triggered YAP phosphorylation, but this effect was largely demolished in WWC1‐deficient cells (Figure 8D). Moreover, WWC1 interacted with LATS2, and LATS2 overexpression significantly reduced interaction between JCAD and WWC1, indicating that JCAD competed with WWC1 for LATS2 binding (Figures 8E and S8E). To further investigate the precise sites mediating JCAD and WWC1 interaction, WWC1 with mutated WW domains and JCAD with mutated PPxY (PY) motif were generated. As shown in Figure 8F, WWC1 mutants without functional WW1, WW2 or both WW domains exhibited significantly reduced protein interaction with JCAD. In support, JCAD mutants without functional PY1, PY2 or both PY motifs also failed to strongly interact with WWC1 (Figure 8G). Moreover, expression of JCAD with PY motif mutations failed to induce YAP dephosphorylation, nuclear localisation and transcriptional activity (Figure 8H–J). Taken together, these data demonstrate that JCAD competed with LATS2 for WWC1 interaction, in a PPxY‐WW‐mediated manner, to inhibit LATS1/2, but enhance YAP activity and function.

## 3 DISCUSSION

JCAD was identified as a critical regulator for liver regeneration in this study. PH was employed to recapitulate patients undergone liver resection or transplanted with small‐for‐size graft. Moreover, shrinkage of liver mass confers an original drive for hepatocellular proliferation, therefore, serves as an ideal model to uncover the effect of JCAD on liver regeneration. Given that the dynamic alternation of JCAD protein with an elevation in consistent with waves of liver regeneration after PH, JCAD is believed to function as a crucial regulator to maintain liver homeostasis in response to its mass loss. To study the function of JCAD during liver regeneration, JCAD‐KO and *Jcad^△Hep^* mice were utilised. Global and liver‐specific JCAD deficiency markedly impeded liver regeneration post PH, as evidenced by decreased expression of cell cycle‐associated genes and impaired DNA replication. JCAD loss in hepatocytes led to a prolonged cell cycle due to G2/M transition blockage. In contrast, overexpression or replenishment of JCAD in JCAD‐KO primary hepatocytes reversed this phenomenon, which was abrogated by addition of YAP inhibitor, VP. All together, these findings suggest that JCAD might facilitate hepatocellular regeneration in a cell cycle‐dependent manner, which enables to further investigate how JCAD accelerates hepatocellular proliferation in cycle transition at molecular levels.

Numerous studies revealed that the Hippo–YAP signalling pathway participates in cell cycle transition both in vitro and in vivo. Mice with hyperactive YAP signalling were protected from acetaminophen (APAP)‐induced liver damage by reducing expression of genes required to metabolise APAP into its cytotoxic metabolites.^15^ Pharmacological inhibition of MST1/2 enhanced liver regeneration, whilst knockdown of YAP by siRNA or liver‐specific knockout of YAP/TAZ yielded an opposite effect.^15^, ^17^, ^26^ These studies collectively argued that liver regeneration was well regulated by the canonical Hippo–YAP signalling pathway. LATS kinases and the downstream effector YAP, oscillate during cell cycle, whilst the cell cycle governor, anaphase‐promoting complex/cyclosome (APC/C)^Cdh1^ E3 ubiquitin ligase complex, prompted LATS degradation; therefore, put YAP/TAZ under cell cycle control.^18^ In consistent with these previous studies, it was observed that YAP activity was highly illustrated in G1 and G1/S phase in the present study. YAP was hyperphosphorylated during G2/M phase via interacting with the G2/M phase checkpoint kinase CDK1.^19^ Moreover, phospho‐mimetic YAP induced mitotic abnormalities, which intrigued to investigate whether JCAD facilitates cell cycle transition in a YAP‐dependent manner. Thereafter, to investigate the function of JCAD in the Hippo–YAP signalling pathway, gain‐of‐function and loss‐of‐function approaches were utilised, and the findings confirmed that JCAD was an upstream regulator of this critical signalling pathway in the control of cell cycle transition.

It was reported that JCAD harbours two canonical ‘PPxY’ motifs separated by two residues, and binds strongly to WW tandems in WWC1 (*K*_d_ ∼58 nM). Although YAP also harbours two WW tandems as well, JCAD may be unable to bind to YAP in the presence of WWC1 due to the modest affinity of YAP WW tandem to PY motif.^20^ Moreover, WWC1 functions in chromosome alignment and microtubule organisation during mitosis,^27^ and was phosphorylated by mitotic kinases Aurora and CDK1.^28^, ^29^ Therefore, the interactions between JCAD, LATS2 and WWC1 were further investigated. WWC1 functions as a scaffold by binding directly with both SAV1 and LATS1/2, bringing LATS1/2 and SAV1–MST1/2 complex in proximity, and facilitates LATS1/2 phosphorylation by MST1/2, hence, serves as a key step in transduction of Hippo–YAP signals.^21^ In the present study, JCAD was found to directly interact with WWC1, which was mediated by PPxY on JCAD and WW domains on WWC1, respectively. The interaction between JCAD and WWC1 mitigated the interaction between WWC1 and LATS kinases, hence, interfered with LATS1/2 activation by phosphokinases, and led to YAP activation and subsequent cell cycle transition. In concordance with our previous study that JCAD directly inhibits LATS2 kinase activity,^8^ the findings further expanded our understanding of JCAD interplay in the core machinery of the Hippo–YAP signal pathway.

Interestingly, it was observed that JCAD‐KO mice presented a more robust phenotype than *Jcad^△Hep^* mice. As a connection protein, JCAD was initially characterised as cell–cell junction‐associated protein in endothelial cells and JCAD increased the risk of vascular disorders including atherosclerosis,^24^, ^30^ coronary artery disease^31^ and thrombosis.^7^ Given that the entire portal flow through relatively narrowed paths generates shear stress on liver sinusoidal endothelial cells (LSECs) and subsequently increased local paracrine of regenerative factors,^32^ JCAD may be also critical for LSECs for the reason that JCAD expression was regulated by laminar flow,^30^ and give rise to a possibility that shear stress posing on JCAD‐KO LSECs might partially account for the difference of JCAD‐KO mice and *Jcad^△Hep^* mice post PH. Considering that JCAD‐positive cells are mainly located in the portal tracts, the function of JCAD in cholangiocytes and its contribution to hepatic regeneration warrant further investigation. Moreover, further investigations are required to study the underlying effect of JCAD in different models of acute or chronic liver injury.

For the implication of the activation in JCAD–Hippo–YAP signalling axis in liver regeneration, LDLT or small‐for‐size transplantation is a clinical condition to investigate. However, clinical specimens after transplantation are less accessible due to ethic issues. As a tight junction protein, JCAD cannot be secreted into blood therefore limited us to investigate the JCAD expression in patients after liver transplantation. Liver failure is another clinical condition to study the function of JCAD in liver regeneration; however, liver biopsy is contradictory for the reason that malfunction of coagulation in patients with liver failure. Therefore, clinical translation of this study warrants further investigation. To comprehensively clarify how JCAD functions in the liver under pathophysiological conditions, the JCAD–Hippo–YAP signalling axis has been actively investigated on other liver disorders, including liver fibrosis^33^ and cholestasis in our ongoing studies, in which Hippo–YAP pathway is actively involved.

To summarise, this study revealed that JCAD deficiency delays the peak of proliferation due to cell cycle arrest but not abolish overall liver regenerative capacity post PH. JCAD interacts with WWC1, which blocks Hippo signalling transduction, leading to YAP activation and cell cycle progression. In the future, pharmacological and genetic approaches for JCAD modulation might be promising to improve the outcome post liver transplantation.

## 4 MATERIALS AND METHODS

### 4.1 Animal experiments

Global JCAD‐KO mice were a generous gift from RIKEN Center for Life Science Technologies, as reported.^9^ *Jcad^flox/flox^* mice and liver‐specific JCAD‐KO (*Jcad^flox/flox^*, albumin‐cre^−/−^, short for *Jcad^△Hep^*) mice were generated in Shanghai Model Organisms Center, Inc. All mice were housed ad libitum under the specific pathogen‐free facility at Fudan University. For PH surgery, two‐third of the liver was removed as previously reported.^34^ In brief, the left lateral and median lobes were resected. Mice were ethically sacrificed and remnant liver tissues were harvested for subsequent evaluation. Detailed information was provided in Supporting Information.

### 4.2 In situ EdU labelling and immunofluorescent staining

To determine liver regeneration, EdU (100 mg/kg) was injected intraperitoneally 4 h before sacrifice. EdU staining was performed according to the BeyoClick™ EdU proliferation kit guidelines. EdU‐positive cells were counted for five fields (magnification ×100)/animal with ImageJ, and expressed as positive cells per high‐power field. The positivity of immunofluorescent staining was evaluated semi‐quantitatively in a blind fashion. For immunofluorescent staining, briefly, frozen liver sections were incubated with primary antibodies at 4°C overnight and then incubated with fluorescent probe‐conjugated secondary antibodies for 2 h at 37°C. Nuclei were counterstained with 4′,6‐diamidino‐2‐phenylindole.^35^

### 4.3 WB analysis and immunohistochemistry staining

The detailed procedure of WB analysis has been described previously.^36^ Briefly, homogenised liver tissue was subjected to sodium dodecyl sulfate‐polyacrylamide gel electrophoresis (SDS–PAGE), followed by transmembrane, blocking, antibody incubation and imaging. For Co‐IP, cell lysates transfected with overexpressed proteins were incubated with Flag‐tagged magnetic beads at 4°C overnight. The beads were washed and boiled for subsequent immunoblotting. For immunohistochemical staining, paraffin sections (5 μm) were deparaffinised, rehydrated, retrieved and blocked, followed by primary antibody incubation, and horseradish peroxidase (HRP)‐based detection. The detailed information is described in Supporting Information.

### 4.4 RNA extraction, qRT‐PCR and RNA‐Seq

High‐throughput sequencing was conducted for RNA extracted from liver specimens collected 2 days post PH, and RNA‐Seq analysis was performed in LC‐Bio Technology Co. The detailed procedure has been previously described and provided in Supporting Information.^37^ Total RNA was extracted from mouse tissue and various cell lines by phenol–chloroform extraction method, and qRT‐PCR analysis was performed.^8^ Primers used for qPCR are listed in Table S2.

### 4.5 Generation of adenoviral vector expressing JCAD

The coding sequence of mouse JCAD was cloned and ligated with pEntry shuttle vector (Invitrogen). The pEntry‐JCAD clone was recombined with the adenoviral vector pAd/PL‐DEST by an LR recombination reaction (Invitrogen), and pEntry and pAd/PL‐DEST vectors were kind gifts from Dr. Jieliang Chen in the Key Laboratory of Medical Molecular Virology, Fudan University Shanghai Medical College. After PacI digestion, the linearised plasmid was transfected into HEK293 cells and amplified for three rounds. The adenoviruses were purified by discontinuous iodixanol gradient centrifugation. The TCID50 of purified adenoviruses was determined by endpoint dilution assay, and the primary hepatocytes were infected with an multiplicity of infection (MOI) at 20−40.

### 4.6 Cell culture and transfection

Hepatoma Huh‐7 cell line and SMCC‐7721 cell line were reserved in our laboratory. Huh‐7‐trans cell line was isolated from Huh‐7 cells as we reported.^38^ siRNAs against JCAD were selected from BLOCK‐iT™ RNAi Designer (https://rnaidesigner.thermofisher.com) according to rank score, and synthesised by Ribobio Technologies. Transfection was performed with Lipofectamine RNAi‐MAX (Life Technologies) according to the reagent instructions. Sequences for siRNAs against *JCAD* or *YAP1* are listed in Table S1.

For establishment of JCAD‐KO cell line, HEK 293T cells were transfected with pLentiCRISPR vectors containing sgRNA targeting JCAD listed in Table S1 in combination with pMD2.G and psPAX2.^39^ For establishment of JCAD‐overexpressed cell line, the pLVX‐IRES‐puro plasmid containing the full length of JCAD fragment cDNA and packaging plasmids were co‐transfected to Huh‐7 and Huh‐7‐trans cell line. Cells were transduced with the lentiviral‐containing medium and selected for single clone.^8^ Oligonucleotides of siRNA duplexes and sgRNA sequences are listed in Table S1. For construction of plasmids with JCAD mutants, myc‐JCAD plasmids with mutations at PY1, PY2, PY1/2 domains were generated by modification of myc‐JCAD using KOD‐Plus‐Mutagenesis kit according to manufacturer's protocol. Primers PY1 (F/R), PY2 (F/R) and PY1/2 (F/R) listed in Table S4 yielded PY1, PY2 and PY1/2 mutant plasmids, respectively.

### 4.7 Synchronisation of cell cycle phases, flow cytometric analysis and live‐imaging of cell cycle by FUCCI system

For G1/S phase block in cell cycle, DTB was conducted. Briefly, after thymidine (2 mM) incubation for 18 h, Huh‐7 cells were released for 9 h, and then treated with thymidine (2 mM) for another 18 h. Cells were synchronised at G1/S phase and released for indicated time points. For mitotic block, thymidine‐nocodazole blockage was conducted. After treatment with thymidine (Sigma) at 2 mM for 24 h, Huh‐7 cells were released for 3 h, and further treated with nocodazole (Sigma) at 100 ng/mL for 12 h, then released at the indicated time points. For flow cytometric analysis, detailed methods are provided in the Supporting Information. For live‐visualisation of cell cycle, the FUCCI system was transfected into Huh‐7 cells as reported,^40^, ^41^ and cell cycle quantification was conducted in a high content live‐cell imaging device (Opera Phenix from PerkinElmer). For observation of dynamic change in cell cycle, FUCCI system‐transfected Huh‐7 cells were cultured in the same high content live‐cell imaging device with CO_2_ for 24 h, and individual images were captured every 2 h.

### 4.8 Dual luciferase activity assays

Huh‐7 cells were co‐transfected with.5 μg of YAP or PY1, PY2, PY1/2 and WT isoforms of JCAD plasmids, or siRNA against JCAD or YAP, together with.5 μg of 8xGTIIC luciferase reporter plasmid, 100 ng of pRL‐SV40 (as transfection control). Cells were lysed 48 h post transfection and assayed according to manufacturer's instructions (Promega). The firefly luciferase activity was normalised to the *Renilla* luciferase activity (*Firefly* luciferase over *Renilla* luciferase) and presented as a ratio in relative light unit as reported previously.^37^

### 4.9 Isolation of primary hepatocytes and treatment with EGF and VP

Hepatocytes were isolated from mice by two‐step collagenase perfusion as previously described.^42^, ^43^ Hepatocytes were plated in plates pre‐coated with type I collagen and cultured in William's E medium for 24 h, followed by treatment with EGF (PeProtech) at 20 ng/mL for another 24 h before harvesting. VP (MedChemExpress, MCE) was administrated at 5 μM following EGF treatment. Primary hepatocytes from JCAD‐KO and WT mice 2 days post PH were isolated for nuclear protein extraction for YAP detection. The detailed information is described in the Supporting Information.

### 4.10 Statistical analysis

The data are presented as mean ± standard error of mean, and were first subjected to normal distribution and homogeneity. Two‐tailed Student's *t*‐test for two groups and one‐way analysis of variance (ANOVA) or two‐way ANOVA with Tukey's test for multiple comparison were conducted. *p*‐Value <.05 was considered as statistically significant (^*^ *p* < .05, ^**^ *p* < .01 and ^***^ *p* < .001), and ns means not significant (*p* > .05). SPSS software (version 19.0; IBM Corporation) was used for statistical analysis.

## AUTHOR CONTRIBUTIONS

Li Zhang, Yong‐Yu Yang and Li Xie performed experiments. Yuan Zhou, Zhonghua Wang, Jia Ding, Zhenxing Zhong, Yuli Wang and Xiuping Liu provided key reagents or performed tissue analysis. Li Zhang and Jian Wu analysed all data and wrote and finalised the manuscript. Fa‐Xing Yu and Jian Wu are responsible for general conception, funding support and overall supervision.

## CONFLICT OF INTEREST STATEMENT

The authors declare they have no competing financial interests associated with conduction of this investigation.

## ETHICS STATEMENT

Animal experiments were performed according to the procedures approved by the Animal Ethic Committee of Fudan University School of Basic Medical Sciences (#20210302‐051) and following the NIH Guidelines of Experimental Animal Handling and Use.

## Supporting information

Supporting Information

## Data Availability

The raw RNA‐Seq data in this study have been uploaded to the National Center for Biotechnology Information's Sequence Read Archive database (https://www.ncbi.nlm.nih.gov/sra) under accession number: PRJNA1003650. The datasets used and/or analysed during the current study are available from the corresponding author upon reasonable request.
