# Supplementary material for: JCAD deficiency delayed liver regenerative repair through the Hippo–YAP signalling pathway
Source: Clin Transl Med. 2024 Mar 21;14(3):e1630. doi: 10.1002/ctm2.1630 (PMC10955226; doi:10.1002/ctm2.1630)
Supplement: Supplementary file 1 — Supporting Information [file CTM2-14-e1630-s001.docx]

**JCAD Deficiency Delayed Liver Regenerative Repair through the Hippo-YAP Signaling Pathway**

Running title: JCAD, Hippo signaling and liver regeneration

Li Zhang^1#^, Yong-Yu Yang^1#^, Li Xie^1#^, Yuan Zhou^1^, Zhenxing Zhong^2^, Jia Ding^3^, Zhong-Hua Wang^1^, Li Xie^1^, Yu-Li Wang^1^, Xiu-Ping Liu^4^, Fa-Xing Yu^2^*, Jian Wu^1,5,6^*

^1^Dept. of Medical Microbiology & Parasitology, MOE/NHC/CAMS Key Laboratory of Medical Molecular Virology, School of Basic Medical Sciences, Fudan University Shanghai Medical College, Shanghai 200032, China;

^2^Institute of Pediatrics, Children’s Hospital of Fudan University, and Shanghai Key Laboratory of Medical Epigenetics, International Co-laboratory of Medical Epigenetics and Metabolism, Institutes of Biomedical Sciences, Fudan University Shanghai Medical College, Shanghai 200032, China

^3^Jing’an Central District Hospital, Shanghai 200024, China;

^4^Department of Pathology and Laboratory Medicine, School of Basic Medical Sciences, Fudan University, Shanghai 200032, China;

^5^Dept. of Gastroenterology & Hepatology, Zhongshan Hospital of Fudan University, Shanghai 200032, China;

^6^Shanghai Institute of Liver Diseases, Fudan University Shanghai Medical College, Shanghai 200032, China

**#**These authors contributed equally.

***Corresponding authors:**

**Jian Wu, MD, PhD, FAASLD**

Department of Medical Microbiology, Key Laboratory of Molecular Virology

Fudan University School of Basic Medical Sciences

138 Yixue Yuan Road, P. O. Box 228

Shanghai 200032, China

Tel: +86(21)5423-7705; Fax: +86(21)6422-7201

Email: [jian.wu@fudan.edu.cn](mailto:jian.wu@fudan.edu.cn)

ORCID: 0000-0001-9933-7364

**Fa-Xing Yu, PhD**

Institute of Pediatrics, Children’s Hospital of Fudan University, and Shanghai Key Laboratory of Medical Epigenetics, International Co-laboratory of Medical Epigenetics and Metabolism, Institutes of Biomedical Sciences

Fudan University Shanghai Medical College

138 Yixue Yuan Road, Research Building 2A, room A3-010

Shanghai 200032, China

Email: [fxyu@fudan.edu.cn](mailto:fxyu@fudan.edu.cn)

ORCID: 0000-0003-4834-9335

This PDF file includes:

Materials and methods

Figures S1 to S8

Tables S1 to S5

**MATERIALS AND METHODS**

Animal models

For global JCAD knockout (KO) mice generation, in short, exon 3 of the *KIAA1462* gene in embryonic stem (ES) cell was replaced by the loxP-flanked neomycin-resistant gene, and targeted ES clones were further transferred to pseudopregnant ICR mice. The offspring were further bred and genotyping by PCR, and primers were listed in Supplementary Table 3. Liver-specific JCAD knockout (*Jcad^flox/flox^*, albumin-cre^-/-^, short for *Jcad^△Hep^*) mice were generated by crossing the *Jcad^flox/flox^* mice, in which exon 3 open reading frame of JCAD was flanked by two flox sequences (generated in Shanghai Model Organisms Center, Inc.), with albumin-cre recombinase transgenic mice. All mice were housed ad libitum under the specific-pathogen-free facility at Fudan University.

Partial hepatectomy (PH)

In brief, age-matched male mice (about 10-12 weeks) were anesthetized with 2% isoflurane. The abdominal cavity was exposed by a midline incision, and after ligation and incision, the left lateral and median lobes were removed. The incision was sterilized after closing the abdominal cavity, and mice were keeped on a warm pad until wake. Mice were ethically sacrificed and remnant liver tissues were harvested, then preserved either with formalin fixation or cutting into pieces and stored in -80 ℃ for subsequent detection.

Western blot analysis and immunohistochemistry staining

Liver tissue was homogenized in radio immunoprecipitation assay (RIPA) buffer, and protein content was quantified by bicinchoninic acid assay (BCA) kit (Thermo Fisher Scientific). Protein lysate was mixed with 5x sodium dodecyl sulfate (SDS) laoding buffer, boiled for 5 min at 100 ℃. After SDS-polyacrylamide gel electrophoresis (SDS-PAGE) separation and transmembrane with polyvinylidene fluoride (PVDF) membrane, proteins were blocked by either 5% milk or 5% BSA, and incubated with primary antibodies at 4 ℃ overnight respectively. Membranes incubated with second antibodies were further imaged with chemiluminescence reagent. For co-immunoprecipitation, cell lysates transfected with overexpressed proteins were incubated with Flag-tagged magnetic beads at 4 ℃ overnight. The beads were washed with ice-cold phosphate buffered saline (PBS) for four times and with ddH_2_O for the last time, and boiled in 5x SDS loading buffer before subsequent immunoblotting. For immunohistochemical staining, paraffin sections (5 μm) were deparaffinized, rehydrated, retrieved and blocked by 3% hydrogen peroxide (H_2_O_2_) and 5% goat serum and incubated with primary antibody overnight at 4 °C. After 1 hour of incubation with secondary antibody at 37 °C, 3,3-diaminobenzidine (DAB) substrate solution was applied to develop the color. Nuclei were counter-stained with hematoxylin. The detailed information of antibodies was described in Supplementary Table 5.

RNA extraction, quantitative reverse transcriptase polymerase chain reaction (qRT-PCR) and RNA sequencing (RNA-Seq)

High-throughput sequencing was conducted for RNA extracted from liver specimens collected two days post PH, and RNA sequencing analysis was performed in LC-Bio Technology CO. (Zhejiang, China). In brief, enriched mRNA was broken into fragments (~200bp) and reversely transcribed into complementary DNA (cDNA), and the cDNA library was constructed from each liver specimen. After amplification and purification, double-end libraries were established and sequenced in an Illumina Novaseq 6000 sequencing platform (LC-Bio Technology). For data processing, HISAT2 (version 2.21), SAMtools (version 1.4) and StringTie (version 2.21) were used to analyze raw data. EdgeR was used to analyze differential gene expression and statistical significance. Total RNA was extracted from mouse tissue and various cell lines by phenol-chloroform extraction method, and qRT-PCR analysis was performed. The specificity of amplification was verified by unique peak of the melting curve, and relative gene expression was analyzed based on the 2^-∆∆CT^ calculation. Glyceraldehyde-3-phosphate dehydrogenase (GAPDH) or β-actin was used as a house-keeping gene control. Primers used for qPCR were listed in Supplementary Table 2.

Flow cytometric analysis

For flow cytometric analysis of cell cycle, single cell suspension at a density of 5×10^5^ to 1×10^6^ was fixed by cold 70% ethanol for 30 min at 4 ℃, washed and resuspended in 250 µl of propidium iodide (PI) (BD Biosciences) and 5 µl of RNase (DNase-free) in the dark at room temperature for 1 hour. DNA content was measured in a flow cytometer from BD Calibur and the resulting data were analyzed with Flowjo. Flow cytometry analysis of apoptosis was performed on these cells using an Apoptosis Detection Kit (Beyotime Biotechnology) based on Annexin V and PI staining.

Isolation of primary hepatocytes

Briefly, after placing a catheter into the inferior vena cava, the portal vein was cut, and the superior vena cava was clamped after chest was opened up. The liver was perfused at 5-7 ml/min with pre-warmed Hanks I perfusion medium for 10 minutes. Then *in situ* dissociation was performed with pre-warmed Hanks I (EDTA excluded) perfusion medium containing Type Ⅳ collagenase and Ca^2+^ at 3 ml/min for 10 min. After dissociation, cells were filtered through a 200-μm filter. Hepatocytes were further separated and purified by Hanks Ⅱ wash buffer (EDTA included) and centrifuged at low speed (50 g, 2 min). Cell viability over 80% was usable.

**SUPPLEMENTARY FIGURES**

**
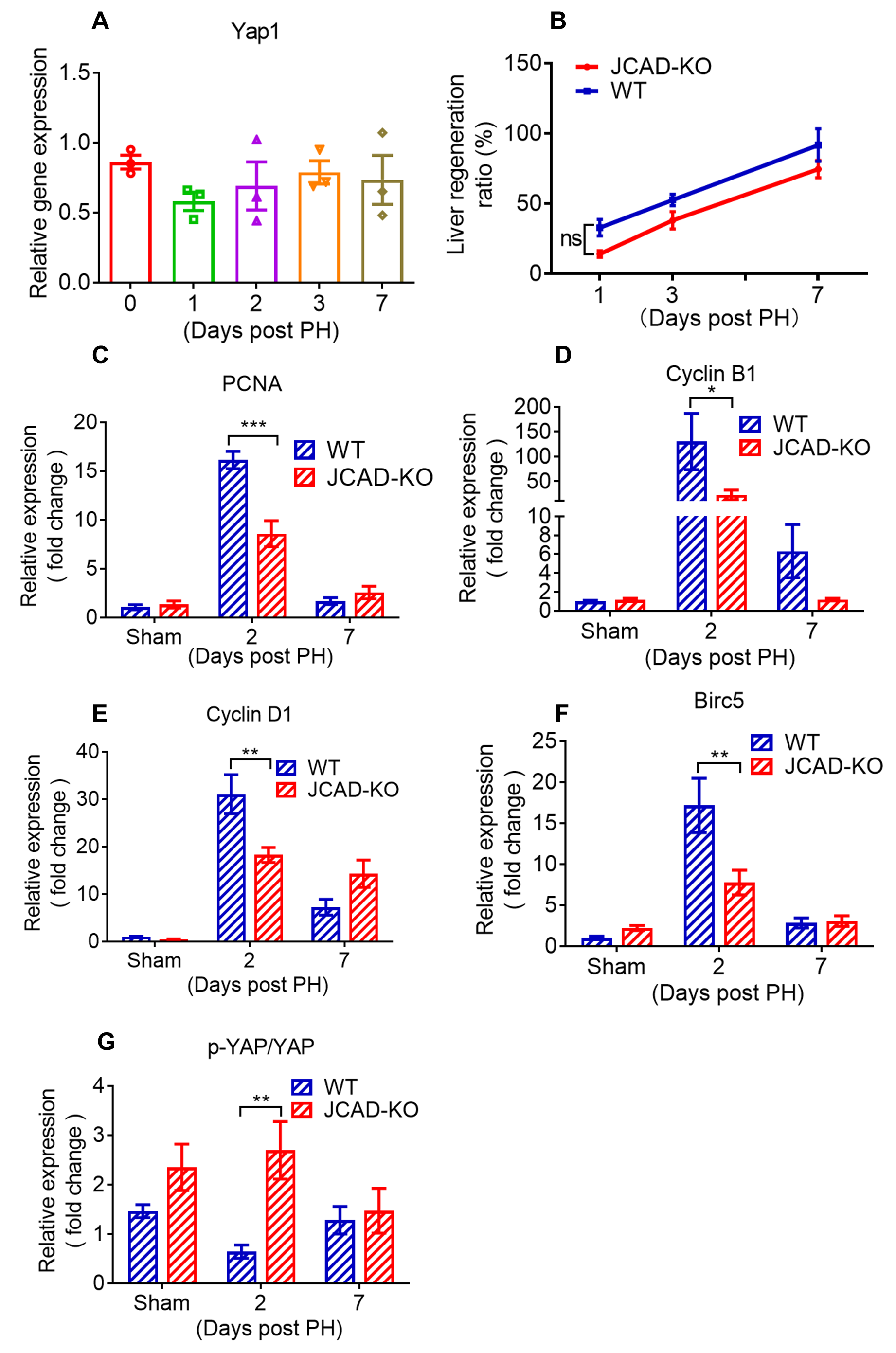
**

**Figure S1. Supplementary data of Fig. 1 and Fig. 2. (A)** Relative gene expression of Yap1 (YAP) was determined by RT-qPCR with β-actin as an internal control (n=3, one-way ANOVA with Tukey’s HSD). **(B)** Liver regeneration index was presented as ST-(TW-RT)/TW, ST: harvested liver residue post PH, TW: calculated liver original weight (RT/70%), RT: removed liver weight (n=6, two-way ANOVA with Tukey’s HSD). **(C-G)** Densitometric quantification of proteins in Fig. 2F (n=4, two-way ANOVA with Tukey’s HSD). All data were presented as mean±SEM. Compared with WT group, *p<0.05, ** p<0.01, ***p<0.001.


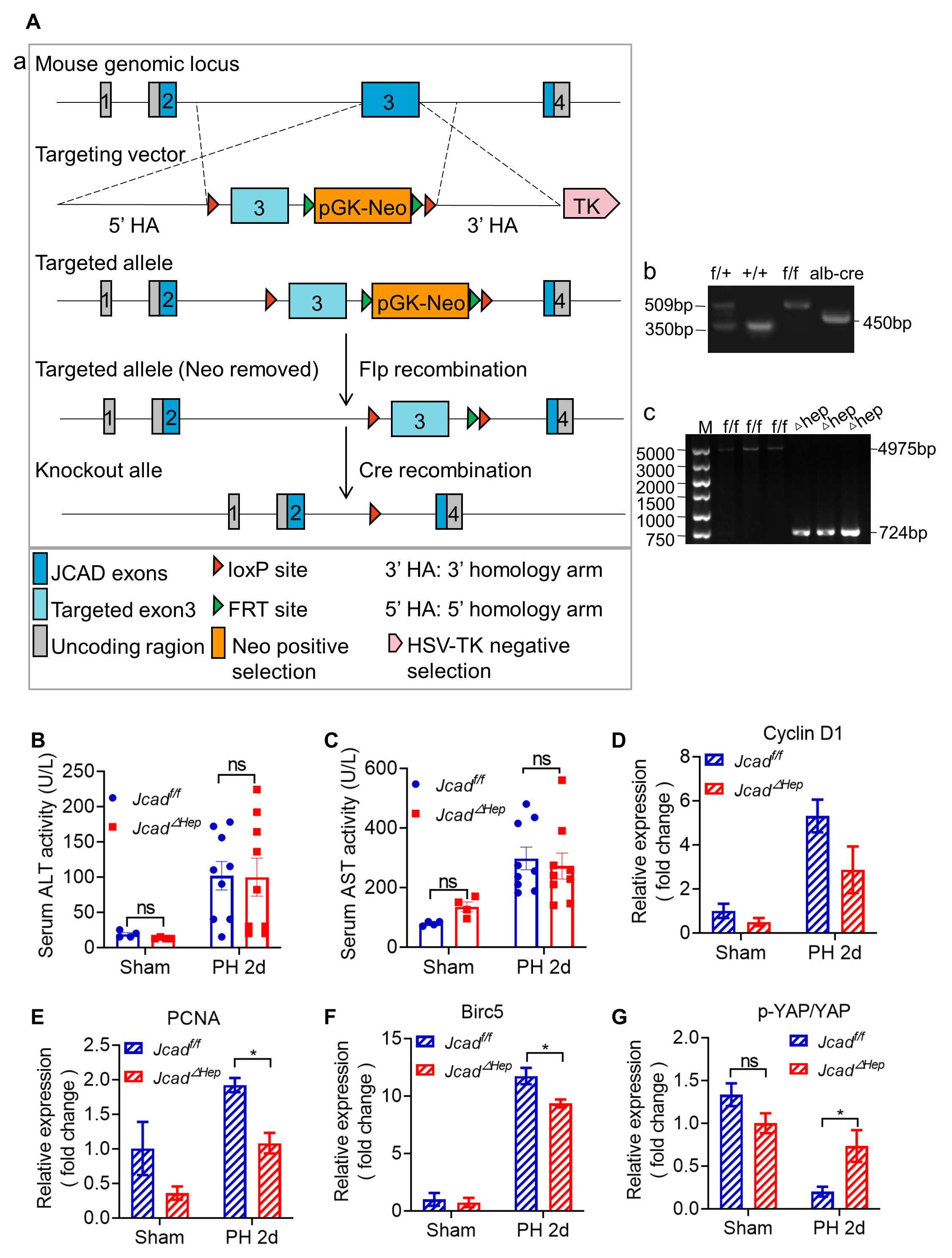


**Figure S2. Genotyping, serum ALT and AST levels and WB intensity of *Jcad^△Hep^*.** **(A) (a)** Schematic representation of the target strategy for the cre-mediated deletion of JCAD exon3. **(b)** Genomic DNA from tail tissue was used for genotyping by PCR analysis. The position of fragments specific for heterozygous floxed (f/+: 509bp, 350bp), homozygous floxed (f/f: 509bp), wild-type (+/+: 350bp), alb-cre (cre: 450bp) are indicated. **(c)** PCR analysis of genomic DNA isolated from liver tissue of individual *Jcad^△Hep^* mouse line. The position of wild-type (f/f: 4975bp) and knockout of JCAD (*Jcad^△Hep^*: 724bp) are indicated. Primers are listed in **Supplementary Table 3**. **(B-C)** Serum ALT and AST levels in *Jcad^△Hep^* mice (n=4 in sham group and n=9 in PH group, two-way ANOVA with Tukey’s HSD). **(D-G)** Densitometric quantification of WB imaging bands in Fig. 3D with GAPDH as a loading control in Figure 2E (n=3, two-way ANOVA with Tukey’s HSD). All data were presented as mean±SEM. Compared with WT group, *p<0.05.


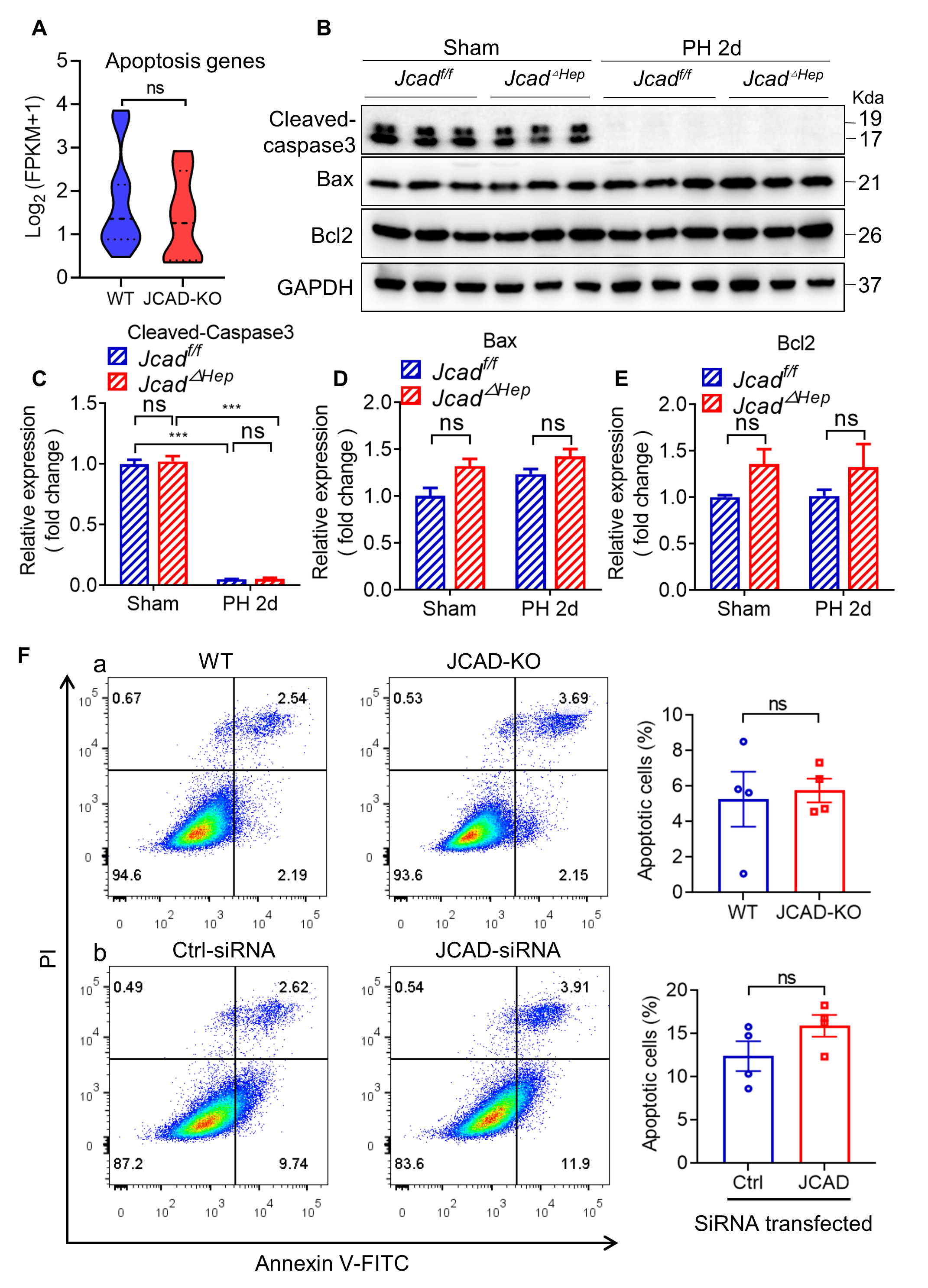


**Figure S3. Apoptosis-associated events were less affected by JCAD knockout during liver regeneration.** **(A)** Apoptosis genes in RNA-sequencing data remained unchanged between WT and JCAD-KO group (n=15 genes for each condition, student’s t test). The median (middle line), 25th and 75th percentile (dot plot) was indicated. **(B-E)** Apoptosis-associated proteins were less affected by JCAD during liver regeneration (n=3, two-way ANOVA with Tukey’s HSD). **(F)** Flow cytometry of PI/Annexin V on JCAD-KO cell line (a) and JCAD siRNA transfected group (b) (n=4, student’s t test). All data were presented as mean±SEM. Compared with sham group, ***p<0.001.


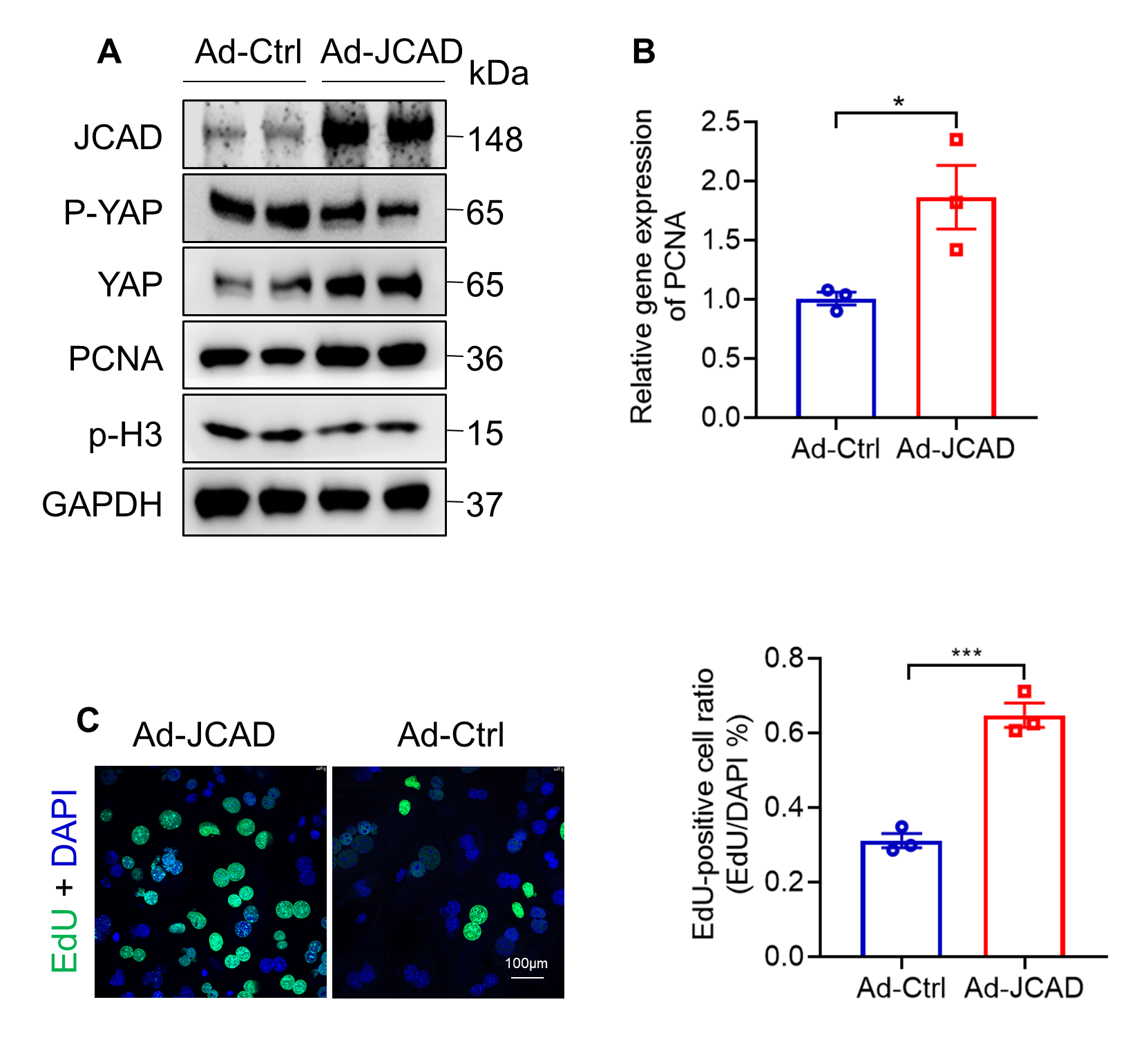


**Figure S4. Validation of JCAD and adenovirus efficacy**. **(A-C)** Adenovirus infection in primary hepatocytes of WT mice. Gene expression **(B)** of PCNA and protein expression of PCNA as well as Hippo signal-related proteins **(C)** were analyzed. EdU incorporation post Ad-JCAD and Ad-Ctrl infection **(A)** were counted, (n=3, student’s t test). All data were presented as mean±SEM. Compared with Ctrl group, *p<0.05, ***p<0.001.


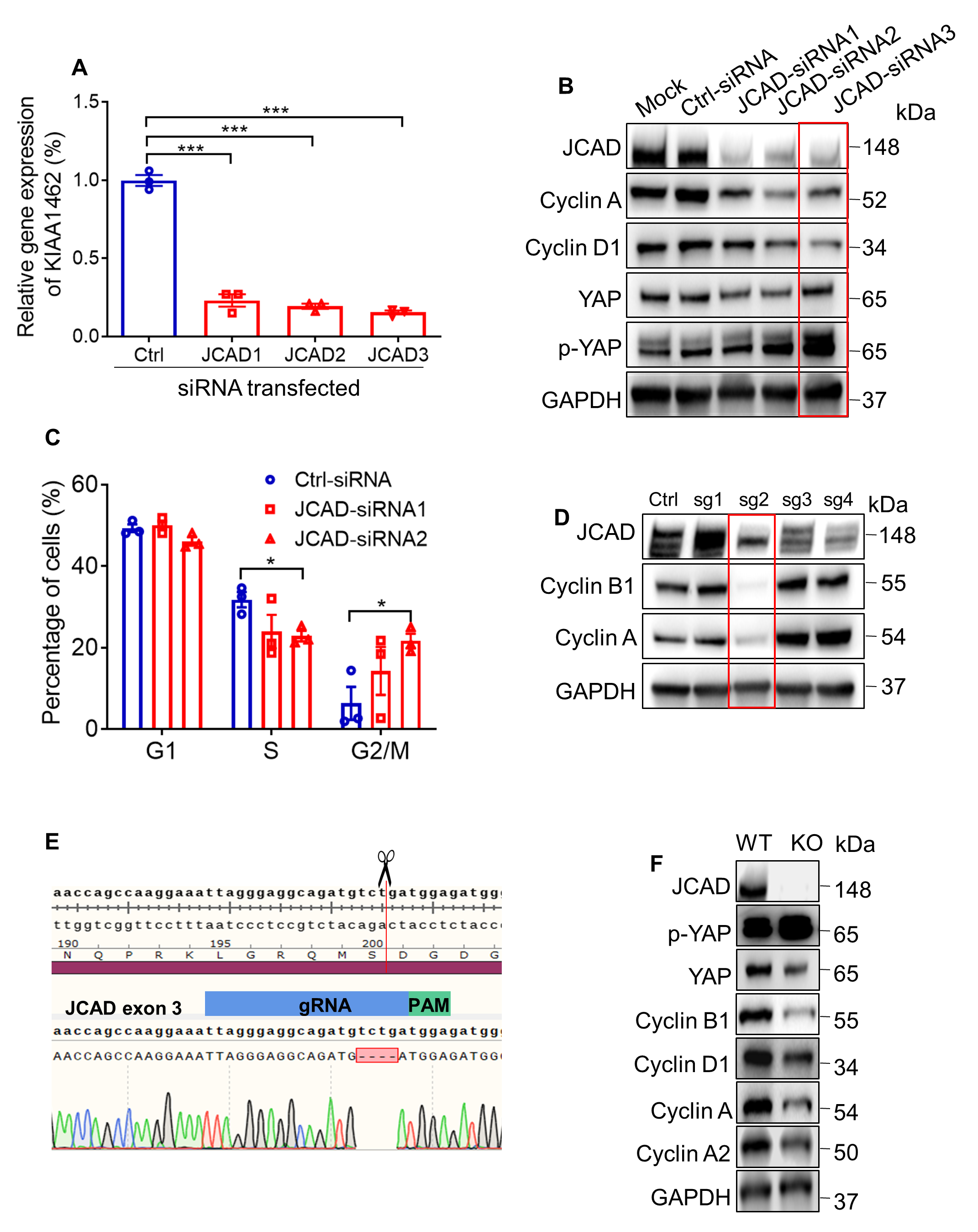


**Figure S5. Transfection efficiency of JCAD siRNA and knockout. (A-B)** Transfection efficiency of JCAD siRNA. Gene expression **(A)** and WB of cell cycle-associated proteins **(B)** were detected after JCAD-siRNA1-3 transfection (n=3, one-way ANOVA with Tukey’s HSD). **(C)** Phase distribution of cell cycle in cells transfected with JCAD-siRNA1-2 was measured by flow cytometry (n=3, two-way ANOVA with Tukey’s HSD). **(D)** Knock-out efficiency of sgJCAD in Huh-7 cells. Cells transfected with lentivirus containing KO2-sgRNA exhibited the most effective in JCAD knockdown. **(E)** Sequencing validation of JCAD knockout in single-clone cells. **(F)** Proteins involved in cell cycle were reduced in JCAD-KO cells. All data were presented as mean±SEM. Compared with Ctrl group, *p<0.05, ***p<0.001.


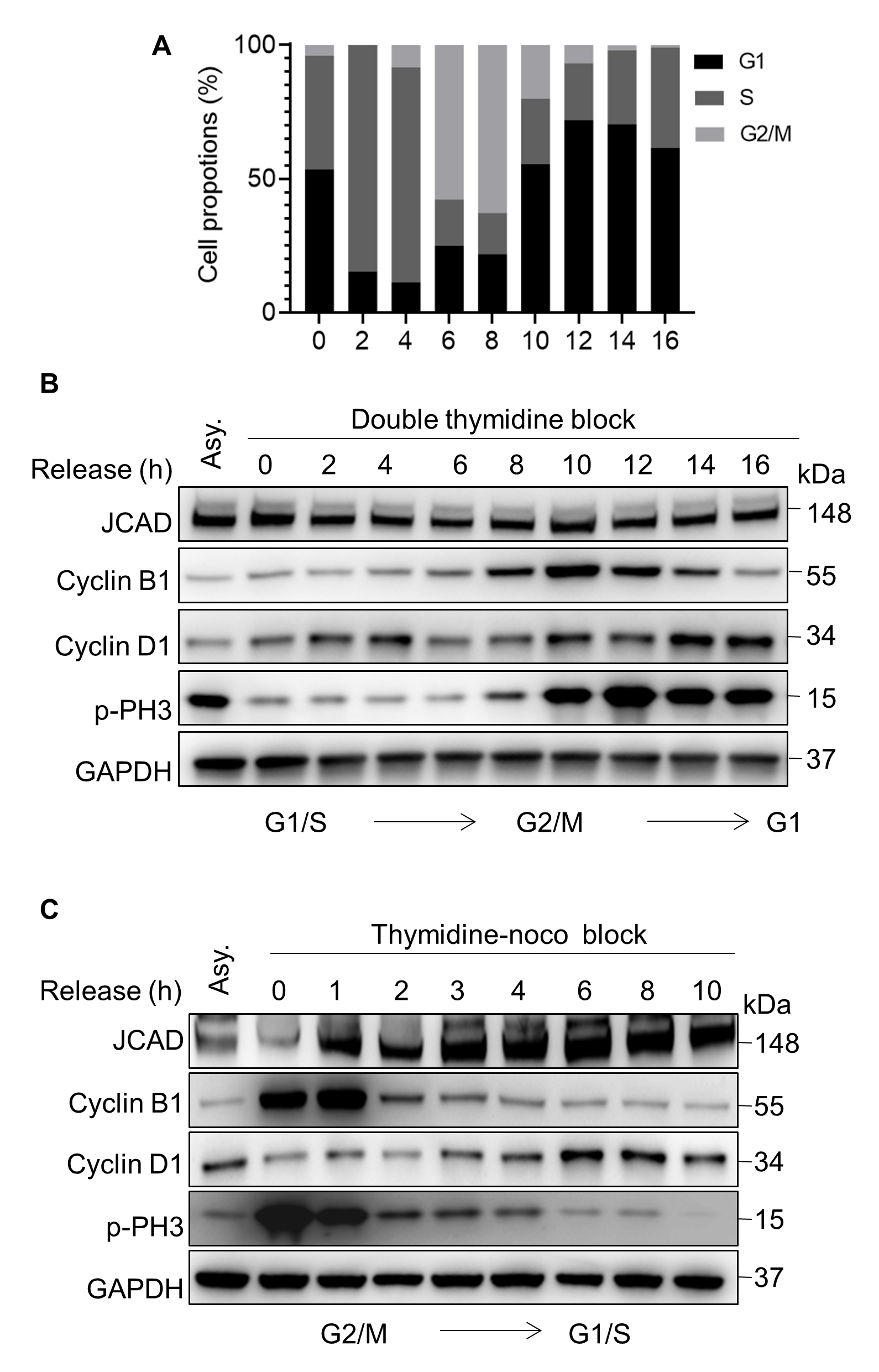


**Figure S6. Cell cycle analysis after double thymidine or thymidine-nocodazole block. (A-B)** Huh-7 cells were synchronized with a double thymidine block, and released for indicated time points. Cell cycle profile was obtained by flow cytometry after PI staining **(A)** and Western blot analysis **(B)**. **(C)** Huh-7 cells were synchronized by thymidine-nocodazole block, and released at indicated time point for Western blot analysis. Asy: asynchronous conditions.


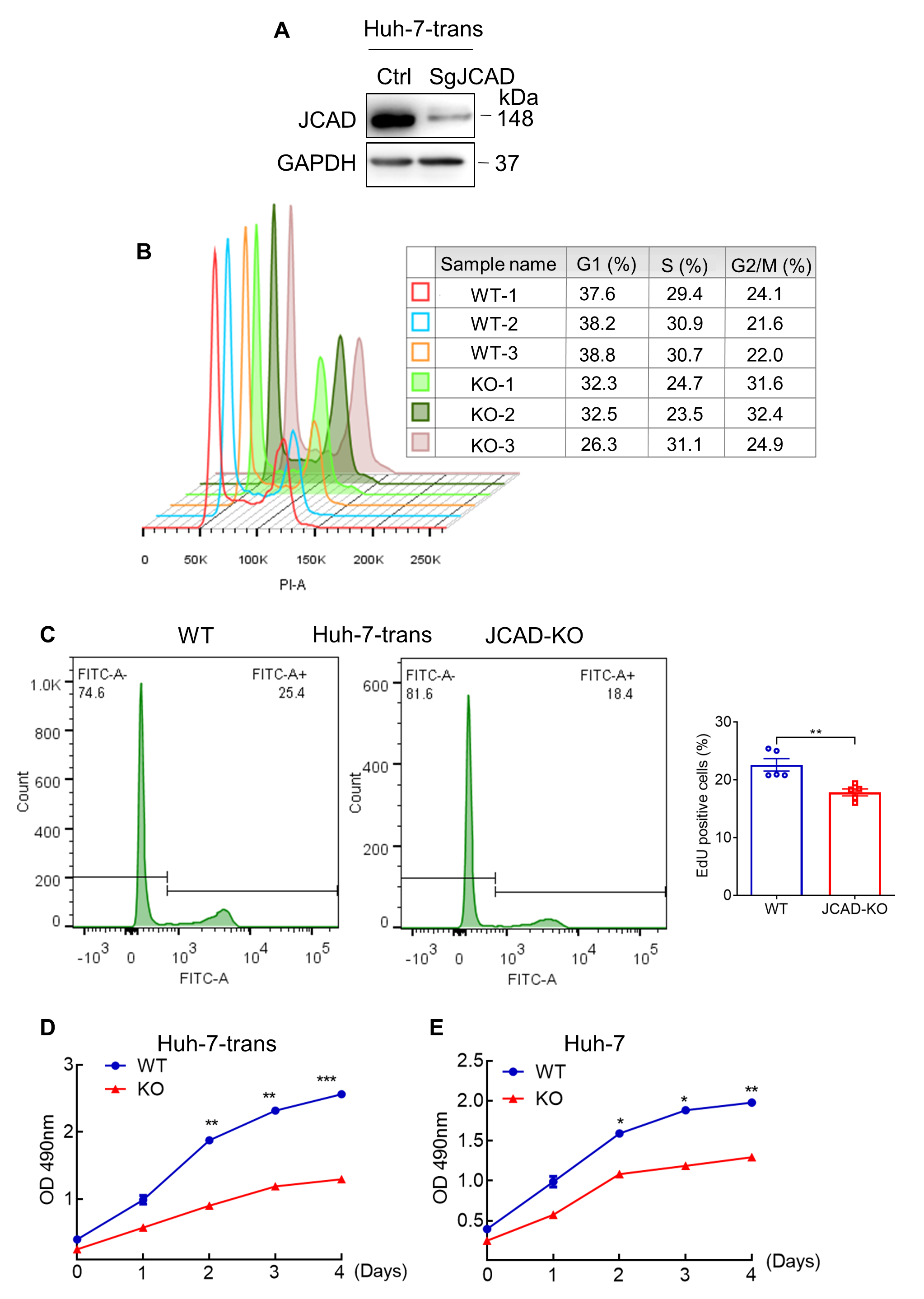


**Figure S7. Flow cytometry and MTT assays of JCAD knockout cell lines.** (A) Knock-out efficiency of sgJCAD in Huh-7-trans cells. **(B)** Cell cycle distribution of sgJCAD Huh-7-trans cells was determined by flow cytometry after PI staining in Huh-7-trans cells. **(C)** EdU-positive ratio of sgJCAD Huh-7-trans cells was counted by flow cytometry (n=5, student’s t test). **(D)** MTT assay of Huh-7-trans and Huh-7 cell lines upon JCAD knockout (n=3, two-way ANOVA with Tukey’s HSD). All data were presented as mean±SEM. Compared with WT cell line, *p<0.05, ** p<0.01, *** p<0.01.


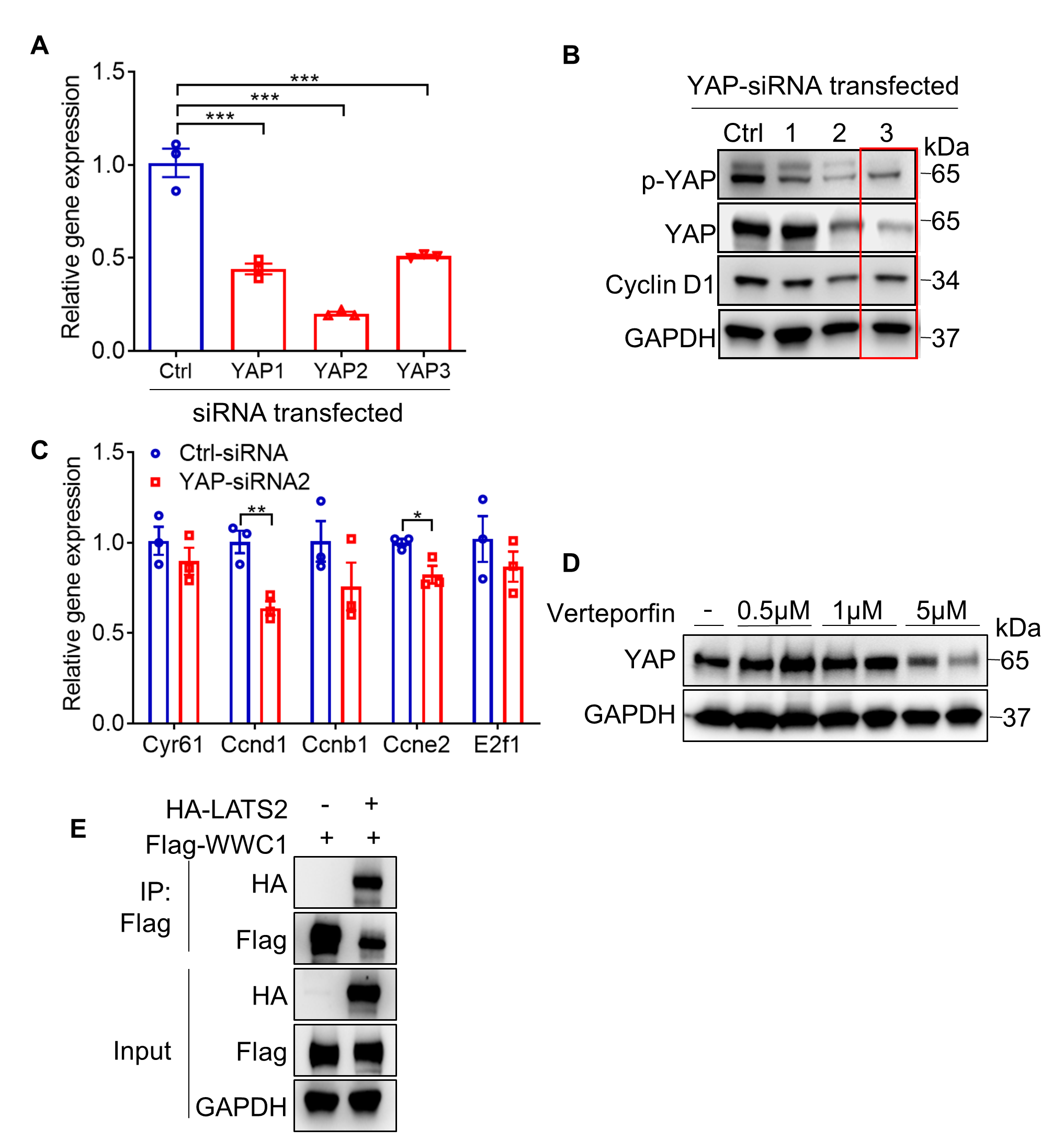


**Figure S8. Efficiency of YAP siRNA, Verteporfin dose and interaction of LATS2 and WWC1 respectively.** **(A-B)** Transfection efficiency of YAP-siRNA. Gene expression **(A)** and WB of cell cycle-associated proteins **(B)** were conducted after YAP-siRNA1-3 transfection, and YAP-siRNA3 was selected for further investigation (n=3, one-way ANOVA with Tukey’s HSD). **(C)** YAP target gene Cyr61 and cell cycle-related gene expression were analyzed in Huh-7 cells transfected with YAP-siRNA2 (n=3, student’s t test). **(D)** Verteporfin dose testing. Huh-7 cells were treated with Verteporfin at 0.5, 1 or 5 μM for 24 hrs then immunoblotting of YAP was conducted. **(E)** Co-IP assay was performed in HEK293T cells transfected with HA-LATS2 and Flag-WWC1. All data were presented as mean±SEM. Compared with cells transfected with Ctrl siRNA, *p<0.05, **p<0.01.

**Supplementary Table 1. Sequences of siRNA and sgRNA**

| **Sequence name** |  | **Sequence (5’-3’)** |
| --- | --- | --- |
| si*GFP*: |  | GTTCAGCGTGTCCGGCGAG |
| *JCAD*siRNA | 1 | GTCAGACCCTGGATTGGAA |
|  | 2 | GCTTGACGATAAATCATAT |
|  | 3 | GAACGGAGTTCCAAAAACA |
| *YAP1*siRNA | 1 | GCGTAGCCAGTTACCAACA |
|  | 2 | CAGTGGCACCTATCACTCT |
|  | 3 | GGTGATACTATCAACCAAA |
| *JCAD*sgRNA | 1 | CACCGCGCACATCGTAAGACGTCCG |
|  | 2 | CACCGAACCGGTCAGCCGTCGTCCC |
|  | 3 | CACCGGGACATAGGCGTCCATCTGC |
|  | 4 | CACCGAATGTCAGGTCCTGCCAAG |

**Supplementary Table 2. RT-PCR primers**

**Mouse**

| **Gene** |  | **Sequence (5’-3’)** |
| --- | --- | --- |
| *Mki67* | F | ACCATCATTGACCGCTCCTT |
|  | R | TTGACCTTCCCCATCAGGGT |
| *Orc1* | F | CTTGTTAGGCCGCAGTCCTC |
|  | R | GGGCTAATGCCACTACCTGA |
| *Cdc6* | F | AAAGGCCCCATGATCGTGTT |
|  | R | CCAATGAGCACCAATCGGGA |
| *Cdt1* | F | CTGGGACCTCTACTCCACCA |
|  | R | CTTGGCCCTTATCCGCTCTA |
| *Ccne2* | F | ATGTCAAGACGCAGCCGTTTA |
|  | R | GCTGATTCCTCCAGACAGTACA |
| *Ccn2* | F | TCCGGACACCTAAAATCGCC |
|  | R | TTCATGATCTCGCCATCGGG |
| *Ccnd1* | F | CCATGGTAGCTGCTGGGAG |
|  | R | CCAGGGCCTTGACCGGG |
| *Yap1* | F | TTCCAACCAGCAGCAGCAAA |
|  | R | ATTCCGTATTGCCTGCCGAA |
| *Wwtr1* | F | GACCCTCATCTCTGGGGGAT |
|  | R | CAGCTCCTTGGTGAAGCAGA |
| *β-Actin* | F | GTCAGAAGGACTCCTATGTG |
|  | R | ACGCAGCTCATTGTAGAAG |
| *E2f1* | F | TGCAGAAACGGCGCATCTAT |
|  | R | CCGCTTACCAATCCCCACC |

**Human**

| **Gene** |  | **Sequence (5’-3’)** |
| --- | --- | --- |
| *KIAA1462* | F | AGACCCTGGATTGGAACCTC |
|  | R | TGACCGCCACACACATTTAT |
| *CCNB1* | F | TGGCCAAATACCTGATGGAAC |
|  | R | ACTTGTTCTTGACAGTCCATTCAC |
| *CCND1* | F | GATCAAGTGTGACCCGGACT |
|  | R | CTTGGGGTCCATGTTCTGCT |
| *CCNE1* | F | AGGGAGCGGGATGCGA |
|  | R | CTCGCCGTCCTGTCGATTTT |
| *CDK1* | F | CCCTTTAGCGCGGATCTACC |
|  | R | CATGGCTACCACTTGACCTGT |
| *CYR61* | F | GATTCGATGCCTCCGAGGTG |
|  | R | GAGGCTCCATTCCAAAAACAGG |
| *GAPDH* | F | TGCACCACCAACTGCTTAGC |
|  | R | GGCATGGACTGTGGTCATGAG |

**Supplementary Table 3. Primers for identification of mouse.**

| **Primer name** |  | **Sequence (5’-3’)** | **DNA size** |
| --- | --- | --- | --- |
| *Alb*cre | F | TGGCAAACATACGCAAGGG | 450 bp |
|  | R | CGGCAAACGGACAGAAGCA |  |
| *Flp* | F | TGGGCAAGTTTGGTGGCTGAG | 4975 bp |
|  | R | GAGGGGAGGGGAGGGAAGA |  |
| *△Hep* | F | CTAGGACTGCAGGGAGAATA | 724 bp |
|  | R | GGAGGGGAGGGAAGAACCATCAG |  |
| *Kiaa* | F | CTCGATCGAGATCCGGAACCCTTAA | 729 bp |
|  | R | GGCCTTCAAGTTCTTCCTGTGTCAG |  |
| WT | F | GGAAGTGTGGCTGGACTGAGAGTCC | 436 bp |
|  | R | GGCCTTCAAGTTCTTCCTGTGTCAG |  |

**Supplementary Table 4. Primers for mutant plasmids construction.**

| **Primer name** |  | **Sequence (5’-3’)** |
| --- | --- | --- |
| PY1 | F | TTCGTGCCTCCGCCCTCATACAGATCGCCCC |
|  | R | CACTGGAGGTTCCAATCCAGGGTCTGACAGGCAGA |
| PY2 | F | TCAGATCGCCCCCGCAGAACATCCCAAA |
|  | R | ATGAGGGCGGAGGCACGTACACTGGA |
| PY1/2 | F | TCAGATCGCCCCCGCAGAACATCCCAAA |
|  | R | ATGAGGGCGGAGGCACGAACACTGGA |

**Supplementary Table 5. Antibodies used in this study.**

| **Antibodies** | **Identifier** | **Source** |
| --- | --- | --- |
| JCAD | ab121545 | Abcam |
| JCAD | NBP1-90941 | Novas Biologicals |
| JCAD | sc-515169 | Santa Cruz |
| YAP | 29495 | Cell Signaling Technology |
| YAP | 13584-1-AP | Proteintech |
| p-YAP | ab76252 | Abcam |
| Ki67 | GB111141 | Servicebio |
| Cyclin D1 | 2922 | Cell Signaling Technology |
| Cyclin B1 | 4138 | Cell Signaling Technology |
| PCNA | sc-25280 | Santa Cruz |
| Cyclin A | sc-239 | Santa Cruz |
| Cyclin A2 | sc-53234 | Santa Cruz |
| p-H3 | AP0002 | Abclonal |
| Cyclin E2 | sc-28351 | Santa Cruz |
| HA-tag | ab9110 | Abcam |
| Flag-tag | M20008 | Abmart |
